# Drug-related risk of severe hypoglycaemia in observational studies: a systematic review and meta-analysis

**DOI:** 10.1186/s12902-015-0052-z

**Published:** 2015-10-12

**Authors:** Marcin Czech, Elżbieta Rdzanek, Justyna Pawęska, Olga Adamowicz-Sidor, Maciej Niewada, Michał Jakubczyk

**Affiliations:** Novo Nordisk Pharma sp. z o.o, Warsaw, Poland; Department of Pharmacoeconomics, Medical University of Warsaw, Warsaw, Poland; Business School, Warsaw University of Technology, Warsaw, Poland; HealthQuest spółka z ograniczoną odpowiedzialnością Sp. K, Warsaw, Poland; Department of Experimental and Clinical Pharmacology, Medical University of Warsaw, Warsaw, Poland; Decision Analysis and Support Unit, Warsaw School of Economics, Al. Niepodległości 162, 02-554 Warsaw, Poland

**Keywords:** Diabetes mellitus, Severe hypoglycaemia, Antidiabetic medications, Insulin regimens, Observational studies

## Abstract

**Background:**

Diabetes mellitus (DM) leads to multiple complications, including severe hypoglycaemia events (SHEs). SHEs can impact a patient’s quality of life and compliance and may directly result in additional costs to the health care system. The aim of this review was to evaluate the risk of severe hypoglycaemia in patients with type 1 (T1) and 2 (T2) DM as observed in everyday clinical practice for various drug regimens.

**Methods:**

We conducted a systematic review of observational (retrospective or prospective) studies in the MEDLINE, Embase, and Cochrane Library databases that covered at least 100 children or adults with T1/T2 DM. In T1 DM, basal-bolus/pre-mix insulin (human or analogue) and insulin pump were reviewed, and in T2 DM, basal-bolus/pre-mix insulin (human or analogue), oral antidiabetic drugs supported with basal insulin (human or analogue), sulfonylureas in monotherapy, and combined oral treatment were reviewed. In order to estimate SHE rates, we extracted data on the time horizon of the study, number of patients, number of SHEs, and number of patients experiencing at least one SHE. We used a random effects model to estimate the annual SHE rate. We considered the risk for other antidiabetic medications in T2 DM to be negligible and the results of our main review yielded no observational data for premixes in T1 DM so they were assessed based on relative rates taken from additional systematic reviews. The study, being a desk research, did not involve any human subjects (including human material or human data) and no ethical committee approval was asked for. For the same reason there was no need to collect informed consent for participation in the study.

**Results:**

We identified 76 observational studies encompassing 707,722.30 patient-years. The estimated annual SHE rate varied from 0.168 (95 % CI 0.123–0.237) for insulin pump up to 1.628 for biphasic human insulin in T1 DM patients, and from 0.0035 for oral antidiabetic drugs up to 0.554 (95 % CI 0.157–7.534) for basal-bolus with human insulin in T2 DM patients.

**Conclusions:**

Our review indicates that SHE rates differ between patients depending on treatment regimen. However, SHEs are also driven by other factors. Proper modelling techniques are needed to use various types of information in published studies.

**Electronic supplementary material:**

The online version of this article (doi:10.1186/s12902-015-0052-z) contains supplementary material, which is available to authorized users.

## Background

Diabetes mellitus (DM) is a common disease with increasing prevalence [1]. DM can lead to microvascular complications, including retinopathy, nephropathy, and neuropathy, as well as macrovascular complications caused mainly by atherosclerotic changes that cause myocardial infarction, stroke, TIA, and peripheral vascular disease. Hyperglycaemia is an important pathophysiological factor in the development of these complications [2, 3] and key to keeping the glucose concentration close to euglycaemic levels in the treatment of diabetes. This is very challenging in clinical practice because of the risk of treatment-induced hypoglycaemia. Hypoglycaemia can be perceived from clinical, physiological, or pathophysiological perspectives through risk factors and preventive measures. Clinical hypoglycaemia is described by the Whipple triad [4]. A plasma glucose concentration < 3.9 mmol/l (<70 mg/dl) with (documented symptomatic) or without symptoms (asymptomatic) is regarded as the threshold for hypoglycaemic events [5]. A severe hypoglycaemic event (SHE) is defined as one requiring the assistance of another person to actively administer carbohydrate, glucagon, or other resuscitative actions. Plasma glucose measurements may not be available during such an event, but neurological recovery attributable to the restoration of normal plasma glucose levels is considered sufficient evidence that the event was induced by a low plasma glucose concentration [5]. Severe hypoglycaemia is common in both types of DM, but less frequent in type 2 (T2). The number of events depends on many factors, including type, duration of diabetes, and types of treatment. The event rates for severe hypoglycaemia in insulin-treated patients as reported in the literature and expressed as episodes per 100 patient-years range from 62 [3] to 320 [6] in type 1 (T1) DM, and from 0 [7] to 73 [8] in T2 DM.

The objective of this meta-analysis was to assess the SHE rate in patients with T1 and T2 DM in real life settings for various anti-diabetic treatment regimens. As data derived from randomized controlled trials (RCTs) have limited external validity and cannot be used directly to reflect everyday clinical practice, we decided to focus on observational studies. One can argue that many confounders associated with usual clinical practice are not appropriately addressed in randomized settings and can affect the risk of hypoglycaemic events.

The present study is an extension of previously published material [9], and here we include additional systematic reviews from the last 2 years. We also used an estimation method that is likely closer to actual clinical mechanisms, i.e., we accounted for some patients having a bigger risk of SHE and that the events may cluster in a subgroup of patients. The current review is supplemented with two additional systematic reviews targeted to identify additional studies because some treatment regimens were not covered by our primary review.

## Methods

### Main systematic review of observational studies

We conducted a systematic review in the MEDLINE, Embase, and Cochrane Library databases in stages. The original search covered observational studies published between 1 January 2002 and 1 October 2012 and was last updated 18 September 2014. In all of the updates, we consistently used the same methodology described in Online Resource Additional file 1 (and in [9]). Although the systematic review did not have a registered protocol, we followed the Preferred Reporting Items for Systematic Reviews and Meta-Analyses (PRISMA) guidelines [10].

We included both T1 and T2 DM patients (children or adults) in our analysis. We used a consistent definition of SHE as an event with low plasma glucose levels that requires help from another person to manage. This definition has been used in numerous publications [11–15] and seems to be an attractive and clinically sound choice for results used in cost-measuring studies because it directly links SHEs to resource use.

We decided to include observational studies in the systematic review in order to more accurately assess SHE rates in real-life settings. Moreover, to account for changes in clinical practice in recent years and the possible impact on treatment-related risk, only recent studies published no more than 10 years ago were included. Taking into account our updates, the earliest studies could have been published on 1 January 2002. To balance the number of studies included and the strength of evidence, we only included studies of some minimal reasonable size, which we defined as at least 100 patients in the total study, though possibly split between several groups.

We designed our review to differentiate between the following treatment regimens: insulin pumps, basal-bolus insulin therapy with long-acting insulin analogue as the basal component (BBA), basal-bolus insulin therapy with human insulin as the basal component (BBH), biphasic insulin analogue, and biphasic human insulin in T1 DM, and sulfonylurea (SU) with or without other oral drugs but excluding insulin, basal long-acting insulin analogue (BOTA), basal human insulin (BOTH), BBA, BBH, biphasic insulin analogue, and biphasic human insulin (all insulin regimens could be in combination with other antidiabetic drugs, OADs) in T2 DM. We defined basal-bolus insulin therapy as long-acting insulin analogue once or twice daily and short/ultrashort insulin at mealtime. In the main systematic review, OADs in T2 DM, especially oral antidiabetic medications different than SU, were neglected assuming that the risk of SHE is negligible. We planned to only assess risk indirectly using information on relative rates from studies searched for in a separate systematic review of secondary studies as described below.

Two authors independently conducted the selection process for relevant trials (basic search: J.P. and E.R.; 1^st^ update: J.P. and E.R.; 2^nd^ update: E.R. and M.J.). Protocol assumed that in the case of discrepancies between the authors, a discussion would be held until consensus was reached. We extracted the following parameters: time horizon at which hypoglycaemia was assessed, number of patients in the study group, number of hypoglycaemic episodes (absolute or mean per patient in a specified period of time, if available), and number of patients experiencing at least one SHE (if available). If one study was described in more than one manuscript, then the manuscript with the most appropriate and complete results was selected for extraction (e.g., data for a total study cohort instead of subpopulation, results presented separately for patients with T1 and T2 diabetes, or results split by insulin regimens of interest). Data from included studies were extracted by one of the reviewers and verified by the other.

We assessed the quality of the observational studies using the Newcastle-Ottawa Scale (NOS) [16], a tool developed for case–control and cohort studies that allows the quality of non-randomized studies (its design, content, and ease of use) to be assessed. Deeks et al. [17] pointed out that this scale is one of the two best for evaluating non-randomized interventional studies and can be used in systematic reviews as either a scale or a checklist. This tool is also mentioned in the Cochrane Handbook as a tool that can be used for assessing methodological quality or risk of bias in non-randomized studies [18]. Thus, in our systematic review we decided to use NOS for case–control and cohort studies, while in order to assess the quality of other types of non-interventional studies we focused on the following elements: patient selection methods, methods for recording outcomes (regarding only severe hypoglycaemia), study size, and study representativeness. According to a recent systematic review of tools used to assess the quality of observational studies [19], there are 97 tools (46 scales and 51 checklists) that can be used to evaluate observational studies, but a transparent objective assessment of the quality of observational research is still missing.

As it was a desk research, the study did not involve any human subjects (including human material or human data) and no ethical committee approval was asked for. For the same reason there was no need to collect informed consent for participation in the study.

### Supplementary systematic reviews

As mentioned above, we planned to assess the risk related to OADs in T2 DM by calculating the relative rates compared to SU based on secondary studies and then impose these relative rates on the background SU-related SHE rate. Our main systematic review also yielded no observational studies for biphasic therapies in T1 DM; therefore, we decided to use similar methodology to assess the SHE rate for that treatment regimen.

In the first of the two additional systematic reviews we used the following approach. We searched MEDLINE, Embase, the Cochrane Library, and Centre for Reviews and Dissemination (CRD) to identify secondary studies (systematic reviews and meta-analyses) that can be used to estimate the relative rates (see search strategy in Additional file 1). Studies were included if the search was performed in at least two databases, including at least one of the above databases, by at least two authors (due to the need to confirm the search results) and if the search strategy was described. RCTs of T2 DM required at least one of the following: dipeptidyl peptidase-4 inhibitor, glucagon-like peptide-1 agonist, OADs such as metformin, and TZD. In addition, severe hypoglycaemia had to be defined as an episode when a patient required help from another person. We used RCTs instead of observational studies because they more commonly provide data on relative rates.

We used the following approach in the second of the additional reviews. The MEDLINE database was searched on 25 February 2015 using the search strategy presented in Additional file 1. Only systematic reviews and RCTs (also those included in systematic reviews) were eligible. Studies performed in patients with T1 DM or T1 and T2 DM (with results presented separately) had to compare patients randomized to a group receiving biphasic insulin analogues to a group receiving biphasic human insulin, a group receiving a basal-bolus insulin regimen, or a group on insulin pumps.

### Data synthesis methods for the main review

The data from included studies vary in structure, i.e., some studies present the total number of episodes in a given group of patients over some time horizon, whereas others present the number of patients who experienced at least one event, and some studies present both. Studies also differ with respect to time horizon. In order to increase the flexibility of the model, we chose the Bayesian approach using the Markov Chain Monte Carlo (MCMC) estimation method implemented in JAGS using R.

We based our model on negative binomial distribution for the following reasons. Firstly, in the previous analysis we used the Poisson distribution as a natural choice to model count data that were available [9]. However, in the present paper we decided to account for the fact that episodes tend to be concentrated in a subgroup of patients; in most of the studies, we observed that fewer patients actually experienced any episode than suggested by a Poisson distribution for a given total number of observed episodes. This observation may stem from some individual predispositions (e.g., lifestyle, genetics, etc.) and would require the introduction of a zero-inflation mechanism, i.e., the fact that many patients will have no events but some will tend to have multiple events.

Secondly, we did not have access to individual patient data (i.e., we only observed the total number of episodes in a study); thus, we needed to work with a distribution that can be aggregated to describe the number of episodes in a cohort. We also needed to be able to account for varying time horizons under plausible assumptions. Thirdly, we wanted the results of the estimation to be easily usable in further modelling, and we did not want the SHE rates to vary over time, which would require knowing the longevity of the treatment, even assuming that the time to first SHE is distributed differently than the time to subsequent SHEs and would be a possible way of introducing the zero-inflation phenomenon.

The negative binomial distribution allows for a clustering effect and can be easily mathematically expressed for groups of patients observed for varying time horizons. We decided to use a mixed fixed-effect and random-effect approach by assuming that the parameter measuring the over-dispersion is fixed across studies for a given treatment regimen while the other parameter impacting the absolute rate was random. This approach allows us to account for heterogeneity between studies, whereas taking both parameters in the random-effect approach yielded unstable results. Non-informative prior distributions were taken.

The specification and JAGS codes are given in the supplementary material (Additional file 2).

As studies usually have only one arm, the SHE rate is estimated separately for the individual classes of drugs. Importantly, the aim of the present study was not to compare drugs, which requires two-arm studies, possibly with randomization, but rather to estimate the absolute rate of SHEs for all of the drug classes separately, accounting for the tendency to prescribe various drugs to patients with varying baseline risks of SHEs (e.g., lifestyle) in clinical practice.

The median values of posterior distributions were used as point estimates. The 2.5 and 97.5 percentiles were used as limits of 95 % confidence intervals (CIs). We used 10,000 iterations as a burn-in phase and then collected every fifth of 50,000 iterations. The estimated parameters of the model allow us to assess the SHE rate (i.e., average number of events per patient-year) and the probability of a given patient suffering at least one SHE over a year.

### Data synthesis methods for the relative rates

Data used for the estimation of relative rates from identified studies were provided in terms of 2×2 tables including the number of SHEs and person-time calculated by multiplying a number of patients and a time horizon for each group. Data given in such a format can be aggregated using the Mantel-Haenszel method, which does not require calculating the value of individual outcomes.

In order to estimate relative rates based on RCTs we used the ‘metafor’ and ‘lme4’ libraries available in R software to estimate the incidence relative rate (IRR) when information on the number of episodes was available. We synthesized data using the rma.mh function available in the ‘metafor’ package. This function fits fixed-effects models to a 2×2 table and person-time data via the Mantel-Haenszel method, which is based on a weighted estimation approach. Zero events are not a problem in this method unless there are no events for one of the two groups. In such cases it is necessary to add a constant (e.g., 0.5) to the number of events [20].

## Results

### Systematic review of observational studies

As mentioned previously, we conducted the systematic review in three stages. The basic literature search yielded 6214 records, and after duplicate removal 5220 articles were assessed by titles and abstracts. We reviewed 526 full texts, 101 of which [6, 21–120] met the inclusion criteria. A total of 55 individual trials were eligible for the analysis. The details of trial identification are given in Additional file 3, and the characteristics of included studies are given in Additional file 4.

For T1 DM we included 21 studies described in 33 articles. Among these studies, 14 provided data on SHEs in patients on insulin pumps (6714.61 patient-years total), 7 on BBA (9656.18 patient-years in total), and 6 on BBH (2881.57 patient-years total). As mentioned previously, we did not find any studies on treatment with biphasic insulin in T1 DM and we carried out a supplementary search for studies on pre-mixed insulin (details are given below). For T2 DM we included 35 studies described in 76 articles. Among these studies, 11 provided data on basal long-acting insulin analogue ± OADs (5347.87 patient-years total), 7 on basal human insulin ± OADs (2142.13 patient-years total), 6 on BBA ± OADs (1456.05 patient-years total), 3 on BBH ± OADs (227.46 patient-years total), 12 on biphasic insulin analogue ± OADs (48,168.49 patient-years in total), 6 on biphasic human insulin ± OADs (2265.87 patient-years total), and 6 on SU ± OADs but excluding insulin (1776.00 patient-years total).

The first update of the basic search resulted in 409 records, which were screened by title and abstract, and duplication removal resulted in 31 new full publications. A total of five articles [121–125] met the inclusion criteria, three of them describing new studies. All identified articles provided data for patients with T2 DM. Three publications presented data on biphasic insulin analogue ± OADs (2501.16 patient-years total), two on BOTA (8084.27 patient-years total), and one on biphasic human insulin ± OADs (505.84 patient-years total). The details of trial identification and the characteristics of included studies are given in Additional files 3 and 4, respectively.

The second update yielded 1727 records (1509 after duplication removal), which were assessed by titles and abstracts. We reviewed 318 full texts, from which 58 manuscripts describing 24 individual trials (18 new studies) [126–183] were eligible for the systematic review. The details of trial identification are given in Additional file 3. The characteristics of included studies are given in Additional file 4.

For T1 DM we included eight studies (seven new studies) described in eight articles, six of them provided data on SHEs in patients on insulin pumps (828.97 patient-years total) and one on BBA (99,804.50 patient-years total) and BBH (72,697.25 patient-years total). The results from one of the identified publications are not included in the systematic review because we previously identified and used another manuscript that presented results on the same patient population.

For T2 DM we included 18 studies (12 new studies) described in 53 manuscripts. Among these studies, six provided data on BOTA (1367.50 patient-years total), one on BOTH (332,525.00 patient-years total), four on BBA (106,938.75 patient-years total), one on biphasic insulin analogue ± OADs (124.20 patient-years total), one on biphasic human insulin ± OADs (119.25 patient-years total), and four on SU (1589.39 patient-years total). Results from the other publications were not used because they presented data on populations already assessed in articles identified in previous searches.

We assessed the quality of the studies using the Newcastle-Ottawa Scale (NOS) [16] for case–control and cohort studies. Other types of observation studies were assessed by focusing on four aspects: i) patient selection methods, ii) methods of recording outcomes regarding severe hypoglycaemia, iii) study size, and iv) study representativeness. Generally, the quality of the studies varied. Among the identified case–control studies, only one scored 5 out of 9 points; other studies were of lower quality: three scored 2 points, three scored 3 points, and two scored 4 points. Among cohort studies, three scored 8 of 9 possible points; other studies were of lower quality: one scored 5 points, eight scored 6 points, and six scored 7 points. The residual studies were of medium quality as assessed by descriptions with no scoring.

### Additional systematic reviews

In order to estimate the expected annual number of SHEs related to OADs (mainly oral) in T2 DM we performed an additional systematic review. This search resulted in a total of 958 potentially relevant publications, which were assessed by titles and abstracts. After duplicate removal, 215 full publications were evaluated, from which 12 systematic reviews fulfilling predefined inclusion criteria were identified (Additional file 3). Of the included systematic reviews, a study conducted by Karagiannis et al. [184] was assessed to provide the most appropriate data on SHEs associated with various antidiabetic medications in T2 DM. We reviewed all studies included in Karagiannis et al. [184] in order to obtain the number of SHEs in each of them. We also verified the definition of severe hypoglycaemia; if it did not comply with the definition assumed for our systematic review, the results from the study were excluded. A total of 11 RCTs [185–195] were identified and included in the analysis.

Our main systematic review yielded no observational studies for biphasic therapies in T1 DM; therefore, we performed an additional systematic review. A total of 454 records were assessed by titles and abstracts, among which 24 full publications were evaluated and 12 met the inclusion criteria. An additional two publications were identified from the references (Additional file 3). A total of seven systematic reviews described five relevant RCTs (see Additional file 3) [196–209]. These studies were then used to assess the relative risk for biphasic therapies in T1 DM.

### SHE rates for treatments based on the main review

The information on the number of SHEs in individual studies is presented in Additional file 5.

The results of the data synthesis are presented in Table 1.

**Table 1:** Annual mean (95 % CI) number of SHEs in patients with T1 and T2 DM

Therapy	Average number of SHEs per patient per year	Probability of ≥1 SHE for a patient annually
T1		
Insulin pump	0.168 (0.123–0.237)	11.38 % (8.09 %–16.03 %)
Basal-bolus (basal insulin analogue)	0.472 (0.252–1.055)	21.37 % (11.30 %–42.97 %)
Basal-bolus (basal human insulin)	1.084 (0.530–2.900)	33.77 % (17.93 %–67.53 %)
T2		
BOT analogue	0.113 (0.050–0.324)	5.55 % (2.32 %–15.62 %)
BOT human	0.173 (0.072–0.600)	7.95 % (3.18 %–26.35 %)
Basal-bolus (basal insulin analogue)	0.080 (0.027–0.456)	4.78 % (1.21 %–27.04 %)
Basal-bolus (basal human insulin)	0.554 (0.157–7.534)	31.40 % (7.44 %–99.64 %)
Pre-mix insulin analogue	0.092 (0.052–0.186)	6.23 % (3.41 %–12.49 %)
Pre-mix human insulin	0.299 (0.137–0.868)	12.43 % (5.87 %–31.85 %)
Sulfonylureas	0.045 (0.023–0.115)	3.57 % (1.91 %–7.56 %)

*BOT* Basal therapy combined with oral antidiabetic medication, *SHE* Severe hypoglycaemia event, *T1, T2 DM* Type 1, Type 2 diabetes mellitus

### SHE rates for treatments based on supplementary reviews

Because the results indicated no significant difference between basal human insulin in a basal-bolus regimen and biphasic insulin analogues (IRR_FE_ = 0.5000, 95 % CI 0.1250–1.992) and the confidence interval is very wide, we assumed that these two treatments are related to an identical SHE rate. The difference between biphasic human insulin and biphasic insulin analogue (IRR_FE_ = 1.5015, 95 % CI 0.9571–2.3558) was also not significant, but the 95 % CI clearly moved towards values >1. The direction agreed with a general tendency of human insulin being related to a greater SHE rate in T2 DM; thus, we used the point estimate to correct the SHE rate compared to biphasic insulin analogue.

For OADs, i.e., metformin, DPP-4, GLP-1, and TZD, the risk of SHEs relative to SU were assessed using data from a previously identified systematic review [184] and referred the risk of SHEs for SU estimated in our systematic review of observational studies. The estimated relative rate for DPP-4 inhibitors and SU was 0.0783 (95 % CI 0.0284–0.2161). There was no significant difference in the risk rate between other OADs and GLP-1. Thus, we applied the relative rate to correct the SHE rate estimated for SU and use it for OADs.

The results are presented in Table 2. Because the average rates of SHE in this case are only based on indirect reasoning, we present no CI and assessed no probability of at least one event.

**Table 2:** Annual mean number of SHEs in patients with T1 and T2 DM

Therapy	Average number of SHEs per patient per year
T1	
Pre-mix insulin analogue	1.084
Pre-mix human insulin	1.628
T2	
OADs (excl. SU)	0.0035

*OAD*s Other antidiabetic drugs, *SHE* Severe hypoglycaemia event, *SU* Sulfonylurea, *T1, T2 DM* Type 1, Type 2 diabetes mellitus

## Discussion

### Data

In this review, we attempted to assess the real-life risk of SHEs associated with various drug regimens. Data selection was constructed to best fit this goal. Because we wanted to assess risk in everyday clinical practice rather than an experimental setting, we decided to use observational studies and not RCTs. Moreover, many factors can change over time, such as clinical practice in treating DM, patient awareness, and lifestyle. Therefore, we decided to focus only on newer studies. As our systematic review was performed in three waves, our data selection encompassed the period starting 1 January 2002 and lasted no more than 13 years. We decided to disregard small studies, assuming that they would contribute little to the total information and that a smaller study size could potentially be associated with lower quality. Importantly, these decisions were made to fit the body of evidence to the goal of the study.

Not surprisingly, using observational studies resulted in significant heterogeneity, which we tried to reduce with a consistent definition of SHEs. We decided to use a definition that relates this event to resource consumption, as it makes the results of our study useful in subsequent economic evaluations. As much as the heterogeneity poses quantitative difficulties, it is unavoidable because the population of diabetic patients is heterogeneous when we account for treatment duration, compliance, and lifestyle, among other factors, which may result in very different risks of hypoglycaemia. In this sense it would be naïve to expect homogeneous results. This heterogeneity has been widely observed in published studies of different sizes and designs [3, 6–8]. In order to account for the heterogeneity we used a (partially) random-effect model, and the heterogeneity results in wide confidence intervals, which should simply be treated as an unavoidable price to pay.

We also tried to limit the impact of heterogeneity by splitting the drugs into treatment groups that seem to be clinically related to various SHE rates. The difficulty is that we can expect reverse feedback, i.e., patients with a high risk of hypoglycaemia may use drugs that cause hypoglycaemia less often, and the net effect may be weakened or even reversed.

We considered performing a meta-regression but ultimately decided that the number of studies is too small for most of the treatment regimens. Furthermore, our goal was not to understand other factors impacting the risk of SHEs, but to construct a set of parameters relating the risk to treatment groups used in clinical practice. Applying more complex models in subsequent studies aiming to assess the number/burden of SHEs in some populations would be more difficult because it would require knowing the values of other explanatory variables to input them in the model.

Some of the inclusion criteria help reduce heterogeneity. Using newer studies helps us focus on mechanisms that most likely prevail in present clinical practice. Using larger studies reduces randomness. Excluding studies comes at the cost of making the body of evidence small, but we ultimately decided that this compromise is worthwhile.

The included observational studies were generally case-control and cohort studies of medium and good quality, respectively, indirectly due to the use of the NOS [16], which can be applied only to these two types of studies. However, the majority of included studies could not be classified to either group and were assessed by descriptions with no scoring; the results suggest that they are also of medium quality. According to Shamliyan et al. [19] there are 97 tools (46 scales and 51 checklists) that can be used to evaluate observational studies, but transparent objective assessments of the quality of observational research are missing. We decided to use NOS because it is one of the two best and recommended tools to evaluate non-randomized interventional studies [17].

Ultimately, we had to use different types of studies to assess the risk of OADs in T2 DM and biphasic insulin in T1 DM. The former was part of the methodology assumed from the very beginning and resulted from our conviction that the risk associated with OADs is so small that any possible errors will be small in absolute terms; the latter resulted from the limited availability of observational data. Using data from RCTs violates our general methodology. Also, taking into account the lack of significance in both comparisons for biphasic insulin in T1 DM, other quantitative estimation approaches could be used. This part of our results should be treated with caution, but we still wanted to end up with a set of parameters.

### Methodology

As mentioned above, the present research is an update of a previously published systematic review [9]. We think that the data synthesis was markedly improved in the present version. Using the binomial distribution allowed us to take into consideration that some patients may have greater risk of hypoglycaemia than others and that the events may cluster in some patients, but this distribution does not force this clustering. Estimation results confirm that this is the case, i.e., the estimated parameters show that this phenomenon occurs. For example, for insulin pumps in T1 DM, the average annual number of SHEs is 0.168. Assuming that a Poisson distribution governs the number of episodes in individual patients, we would conclude that 15.5 % have at least one event per year. Using the negative binomial distribution allows us to estimate this parameter separately, yielding 11.4 % instead.

Different approaches have been suggested and were tested by us. For example, a two-step model could be constructed in which a patient is randomly determined to not have any or to have at least one SHE, and then the actual number of SHEs is randomly determined. An alternative method is assuming that the time to the first SHE is distributed differently than the time to the subsequent SHEs. Such approaches would result in more complicated modelling, and it may be difficult to specify the model with no individual patient data and to subsequently use it because it would require knowing the treatment history and information on past SHEs.

Using the negative binomial distribution allows us to account for (but not enforce) the clustering effect and to estimate two parameters of interest: the annual number of events and the risk of experiencing at least one event. The former may be more useful in subsequent economic studies. The latter may be important when analysing the fear of hypoglycaemia attributed to individual treatment regimens.

### Results

From a clinical perspective the results are consistent with the general overview of treatment regimens and associated risk of SHEs. We generally observed that the risk of SHEs is higher for T1 DM than T2 DM and mainly attributable to insulin injections; SU-based oral antidiabetic treatments were found to be related to the lowest risk of SHEs. Insulin analogues are related to a much smaller risk than human insulin, especially in basal-bolus therapy in T2 DM, and pre-mixes and BOTs seem to be related to a reduced risk compared to more intensive treatment with basal-bolus of human insulin (in T2 DM), but not for insulin analogues. The only one striking, but not unexpected, finding is the difference in SHE risk for insulin analogue and human insulin components of basal-bolus therapy in T2 DM. One could argue that we were not able to fully control for diabetes duration and other cofounders, which results in a much higher risk of SHEs attributable to human insulin. Nevertheless, the main and most valuable finding of our study is the quantitative estimation of consistently defined and reported SHE risk related to the most common treatment regimen rather than individual drugs.

### Limitations

This study obviously has numerous limitations. We consider the heterogeneity of the studies to be the most important limitation. Even though heterogeneity was to be expected and is unavoidable, it results in wide confidence intervals; thus, the actual rates may be substantially different than our point estimates. For some treatment groups there were only a few studies, and they may mistakenly present the overall true picture. Nevertheless, the current results try to use the best currently available data.

It is important to correctly perceive the applicability of our results. We did not aim to compare therapies so as to draw interventional conclusions, i.e., so as to conclude about the effect of prescribing this drug instead of another on the risk of SHEs. Because we, in principle, did not use multi-arm studies or randomized trials, that kind of conclusions are not authorized. Thus, there are limitations with respect to the types of questions answered by our study.

Notably, we focussed only on one element related to the risk of hypoglycaemia, but there are many more. We do not claim that the therapy used is the most important determinant of SHE risk. However, this factor can be relatively well quantified and measured and subsequently used to try to estimate the number of SHEs more precisely using strata.

### Relevance to previous research

We performed a simple search in the MEDLINE database to determine if there are other systematic reviews or meta-analyses that evaluate real-life risk of severe hypoglycaemia among patients with T1 and T2 DM for various therapies. We used the search terms “severe”, “major”, “serious”, “hypoglycaemia”, “diabetes”, “observational”, and “real life” to identify potentially relevant citations. We did not find other systematic reviews or meta-analyses that assessed average annual rates of SHEs associated with various insulin regimens and OADs based on observational studies.

A review by Bolen et al. [210] was closest to ours; the aim of their study was to summarize the English language literature on the benefits and harms of oral agents in adult patients with T2 DM. Bolen et al. identified two systematic reviews and 216 controlled trials and cohort studies. All systematic reviews and 167 trials evaluated adverse events, 67 % of which were RCTs and the rest observational. Bolen et al. used a random-effect model to estimate post-treatment differences in absolute risk for adverse events between individual drugs, drug groups, or therapies. Bolen et al. combined results for minor and major hypoglycaemia, whereas our aim was only to estimate the average annual rate of severe hypoglycaemia. The previous review also did not provide a definition of major hypoglycaemia; Bolen et al. [210] conducted a meta-analysis indicating that the use of second generation SU results in a higher frequency of minor and major hypoglycaemia episodes than therapy with metformin or TZD. This trend is in line with our analysis of data from RCTs included in Karagiannis et al., that SU drugs are associated with a higher risk of hypoglycaemia than OADs [184].

Another meta-analysis of observational studies was conducted by Goto et al. [211]. This review evaluated an association between severe hypoglycaemia and the risk of cardiovascular disease in patients with T2 DM based on cohort studies and RCTs, as long as an observational analysis of the analysed association was available. That meta-analysis included six studies: two secondary analyses of RCTs and four based on administrative databases. However, neither of those analyses fulfilled the inclusion criteria of our systematic review due to an inappropriate definition of severe hypoglycaemia. Goto et al. [211] used relative risk as a measure of effect to estimate the association between SHEs and cardiovascular disease. Their findings suggest that severe hypoglycaemia is associated with approximately twice the risk of cardiovascular disease and indicate that an evaluation and quantification of the risk of severe hypoglycaemia is needed.

## Conclusions

Various drug regimens differ in terms of the risk of severe hypoglycaemia and our results are consistent with the general perception of a higher risk of hypoglycaemia being associated with T1 DM compared to T2 DM, insulin-based treatment versus oral antidiabetic drugs, and human insulin versus analogues. Observational studies seem to be well suited for assessing the real-life risk, but they increase the heterogeneity of data. A negative binomial distribution can be used to model various forms of data and allows us to account for clustering, which is an expected clinical phenomena confirmed by our results. The results of our studies can be used to provide parameters for cost-of-illness studies estimating the overall burden of hypoglycaemia.

## Additional files

Additional file 1:
**Search strategies.** (PDF 220 kb) Additional file 2:
**Model specification and JAGS codes.** (PDF 343 kb) Additional file 3:
**Study selection process.** (PDF 318 kb) Additional file 4:
**Studies characteristics.** (PDF 332 kb) Additional file 5:
**Individual studies data.** (PDF 466 kb)

## Abbreviations

BBA: Basal-bolus insulin therapy with long-acting insulin analogue as the basal component
BBH: Basal-bolus insulin therapy with human insulin as the basal component
BOT: Basal therapy combined with oral antidiabetic medication
BOTA: Basal supported oral therapy with long-acting insulin analogue as the basal component
BOTH: Basal supported oral therapy with human insulin as the basal component
CI: Confidence interval
DM: Diabetes mellitus
OAD: Other antidiabetic drug
NOS: Newcastle-Ottawa scale
RCT: Randomized controlled trial
SHE: Severe hypoglycaemia event
SU: Sulfonylurea
T1, T2 DM: Type 1, Type 2 Diabetes mellitus
TZD: Thiazolidinediones
