# Supplementary material for: Drug-related risk of severe hypoglycaemia in observational studies: a systematic review and meta-analysis
Source: BMC Endocr Disord. 2015 Oct 12;15:57. doi: 10.1186/s12902-015-0052-z (PMC4603823; doi:10.1186/s12902-015-0052-z)
Supplement: Additional file 1: — Search strategies. (PDF 220 kb) [file 12902_2015_52_MOESM1_ESM.pdf]

Search strategies

1.1 Systematic review of observational studies – primary search, 1st and 2nd update

The Cochrane Library search strategy	
#1	MeSH descriptor: [Diabetes Mellitus] explode all trees
#2	MeSH descriptor: [Hypoglycaemia] explode all trees
#3	MeSH descriptor: [Sulfonylurea Compounds] explode all trees
#4	MeSH descriptor: [Insulin] explode all trees
#5	MeSH descriptor: [Insulins] explode all trees
#6	diabetes [from 2002]
#7	hypoglycaemia [from 2002]
#8	sulfonylurea [from 2002]
#9	insulin or insulins [from 2002]
#10	Enter terms for search (#1 or #6) and (#2 or #7) and (#3 or #8 or #4 or #5 or #9) [from 2002]

Medline (PubMed) search strategy	
#1	diabetes
#2	hypoglycaemia
#3	sulfonylurea
#4	insulin
#5	insulins
#6	#1 and #2 and (#3 or #4 or #5)
#7	#6 Filters: Comment; Editorial; Guideline; Letter; Practice Guideline; Randomized Controlled Trial; Review
#8	#6 not #7
#9	#8 Filters: Humans, 10 years, English

EMBASE search strategy	
#1	'diabetes'/syn AND ([english]/lim OR [polish]/lim) AND [humans]/lim AND [embase]/lim AND [2002-2013]/py
#2	'hypoglycaemia'/syn AND ([english]/lim OR [polish]/lim) AND [humans]/lim AND [embase]/lim AND [2002-2013]/py

#3	'sulfonylurea'/syn AND ([english]/lim OR [polish]/lim) AND [humans]/lim AND [embase]/lim AND [2002-2013]/py
#4	'insulin'/syn AND ([english]/lim OR [polish]/lim) AND [humans]/lim AND [embase]/lim AND [2002-2013]/py
#5	#3 OR #4
#6	#1 AND #2 AND #5
#7	'diabetes'/syn AND [animals]/lim AND [embase]/lim AND [2002-2013]/py
#8	'diabetes'/syn AND [in process]/lim AND [embase]/lim AND [2002-2013]/py
#9	'diabetes'/syn AND [article in press]/lim AND [embase]/lim AND [2002-2013]/py
#10	'diabetes'/syn AND [randomized controlled trial]/lim AND [embase]/lim AND [2002-2013]/py
#11	'diabetes'/syn AND [metaanalysis]/lim AND [embase]/lim AND [2002-2013]/py
#12	'diabetes'/syn AND [review]/lim AND [embase]/lim AND [2002-2013]/py
#13	'diabetes'/syn AND [conference abstract]/lim AND [embase]/lim AND [2002-2013]/py
#14	'diabetes'/syn AND [conference paper]/lim AND [embase]/lim AND [2002-2013]/py
#15	'diabetes'/syn AND [conference review]/lim AND [embase]/lim AND [2002-2013]/py
#16	'diabetes'/syn AND [editorial]/lim AND [embase]/lim AND [2002-2013]/py
#17	'diabetes'/syn AND [letter]/lim AND [embase]/lim AND [2002-2013]/py
#18	'diabetes'/syn AND [note]/lim AND [embase]/lim AND [2002-2013]/py
#19	'diabetes'/syn AND [cochrane review]/lim AND [embase]/lim AND [2002-2013]/py
#20	'diabetes'/syn AND [systematic review]/lim AND [embase]/lim AND [2002-2013]/py
#21	#7 OR #8 OR #9 OR #10 OR #11 OR #12 OR #13 OR #14 OR #15 OR #16 OR #17 OR #18 OR #19 OR #20
#22	#6 NOT #21

1.2 Systematic review of secondary studies

**The Cochrane Library search strategy**	
#1	MeSH descriptor: [Diabetes Mellitus] explode all trees
#2	MeSH descriptor: [Hypoglycaemia] explode all trees
#3	#1 and #2
#4	#3 and Cochrane Reviews
#5	#3 and Other Reviews
#6	#3 and Technology Assessments
#7	#3 and Economic Evaluation

EMBASE search strategy	
#1	'diabetes'/syn AND mellitus AND [embase]/lim
#2	'hypoglycaemia'/syn AND [embase]/lim
#3	#1 and #2
#4	#3 AND 'systematic review'/de

Medline (PubMed) search strategy	
#1	"Diabetes Mellitus"[Mesh]
#2	Diabetes Mellitus
#3	#1 or #2
#4	"Hypoglycaemia"[Mesh]
#5	Hypoglycaemia
#6	#4 or #5
#7	#3 and #6
#8	#7 AND Filters: Systematic Reviews

CRD (Centre for Reviews and Dissemination) search strategy	
#1	MeSH DESCRIPTOR Diabetes Mellitus EXPLODE ALL TREES
#2	MeSH DESCRIPTOR Hypoglycaemia EXPLODE ALL TREES
#3	#1 and #2

1.3 Systematic review of primary studies for pre-mixed insulins in type 1 DM

Medline (PubMed) search strategy	
#1	biphasic
#2	pre-mix
#3	#1 OR #2
#4	insulin
#5	#3 AND #4
#6	"Diabetes Mellitus"[Mesh]
#7	diabetes
#8	#6 OR #7

#9	type 1
#10	#8 AND #9
#11	insulin dependent diabetes
#12	#10 OR #11
#13	#5 AND #12
