# Supplementary material for: Drug-related risk of severe hypoglycaemia in observational studies: a systematic review and meta-analysis
Source: BMC Endocr Disord. 2015 Oct 12;15:57. doi: 10.1186/s12902-015-0052-z (PMC4603823; doi:10.1186/s12902-015-0052-z)
Supplement: Additional file 2: — Model specification and JAGS codes. (PDF 343 kb) [file 12902_2015_52_MOESM2_ESM.pdf]

## Data synthesis approach in primary studies analysis

Below we present the model specification that was used separately for all analysed treatment regimens. Denote the following variables:

$i$ study index,

$t_i$ duration of study $i$,

$n_i$ number of patients in study $i$,

$r_i$ a parameter of the negative binomial distribution in study $i$,

$p$ a parameter of the negative binomial distribution (identical in all the studies),

$pShe_i$ a probability that a patient experiences SHE during study $i$,

$obsShe_i$ observed number of SHEs in study $i$,

$obsPts_i$ observed number of patients with at least one SHE in study $i$,

$\alpha, \beta$ meta-parameters of the distribution generating $r_i$,

The following model was used:

$r_i \sim \Gamma(\alpha, \beta)$,

$pShe_i = 1 - p^{r_i t_i}$,

$obsShe_i \sim \text{Negative Binomial}(p, r_i t_i n_i)$,

$obsPts_i \sim \text{Binomial}(pShe_i, n_i)$,

$p \sim Beta(1,1)$, (a non-informative prior)

$\alpha \sim \text{Exp}(1)$, (a non-informative hyperprior)

$\beta \sim \Gamma(0.01,0.01)$, (a non-informative hyperprior).

With this specification $\frac{\alpha}{\beta}$ is the expectation of $r$, and so:

$\frac{\alpha}{\beta} \times \frac{1-p}{p}$ measures the expected number of SHEs per patient-year,

$1 - p^{\frac{\alpha}{\beta}}$ measures the probability that a patient will experience a SHE within a year.

Below we present the JAGS code used for calculations.

```
model
{
  for (i in 1 : N) {
    r[i] ~ dgamma(alpha,beta)
    rPatient[i] <- r[i] * t[i]
    rStudy[i] <- rPatient[i] * pts[i]

    pShe[i] <- 1-pow(p,rPatient[i])

    obsShe[i] ~ dnegbin(p, rStudy[i])
    obsPts[i] ~ dbin(pShe[i], pts[i])
  }
    m <- rAvg*(1-p)/p
    prShe <- 1-pow(p,rAvg)
    rAvg <- alpha/beta

    p ~ dbeta(1,1)
    alpha ~ dexp(1)
    beta ~ dgamma(0.01, 0.01)}
```
