# Supplementary material for: Drug-related risk of severe hypoglycaemia in observational studies: a systematic review and meta-analysis
Source: BMC Endocr Disord. 2015 Oct 12;15:57. doi: 10.1186/s12902-015-0052-z (PMC4603823; doi:10.1186/s12902-015-0052-z)

The study selection process.

Figure 1. Systematic review of observational studies (basic search) – the study selection process.

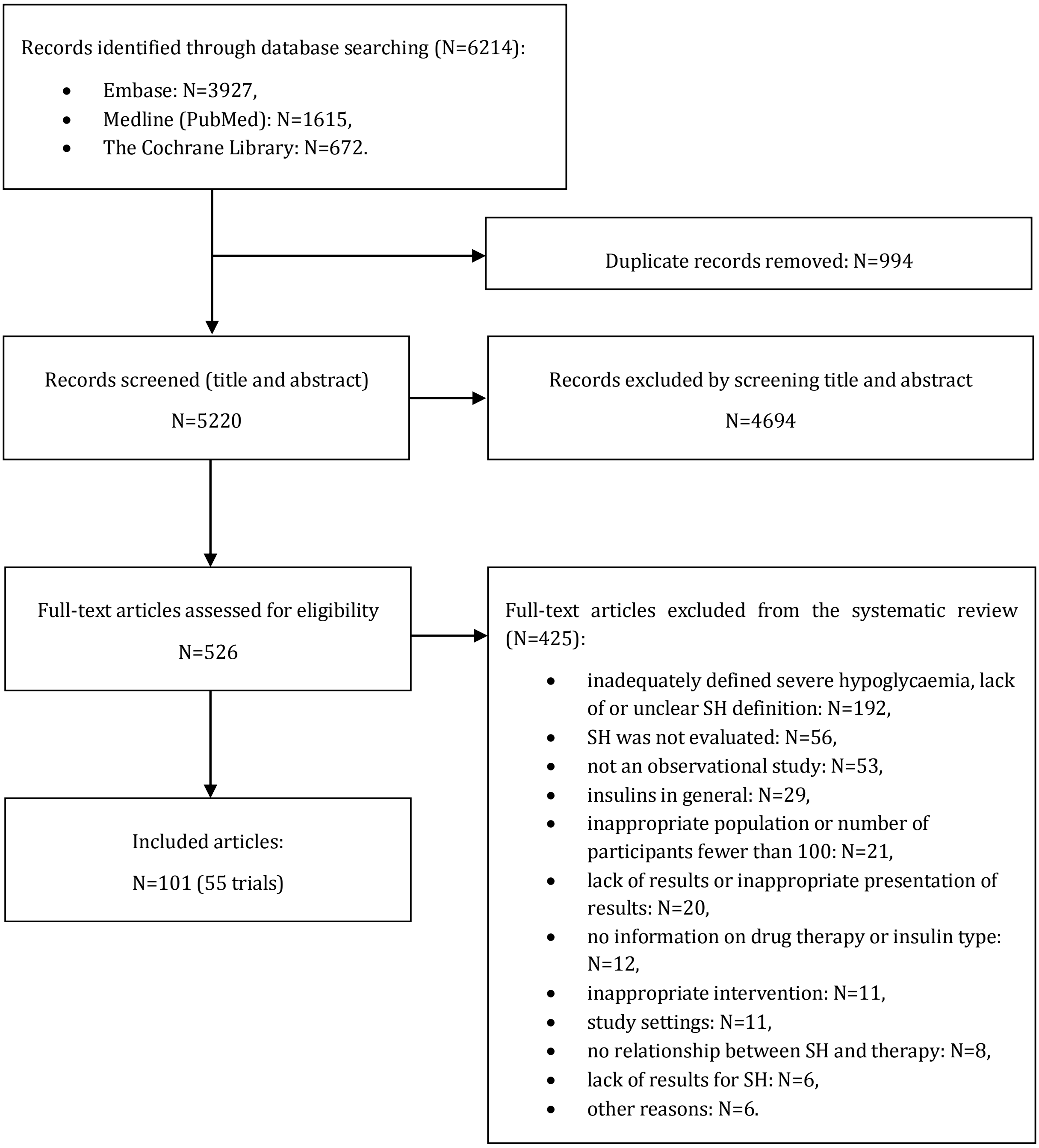

Figure 2. Systematic review of observational studies (1st update) – the study selection process.

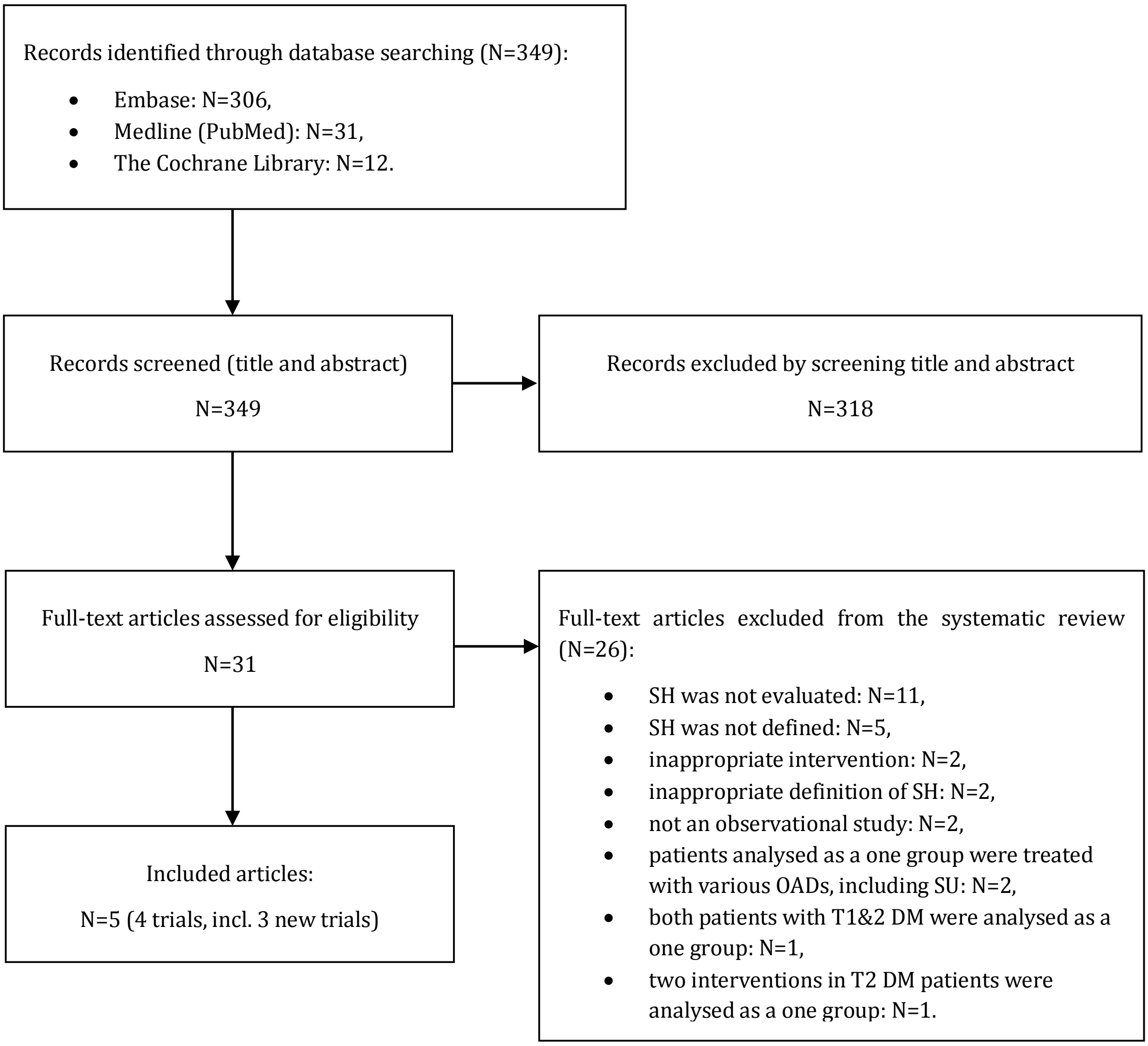

Figure 3. Systematic review of observational studies (2nd update) – the study selection process.

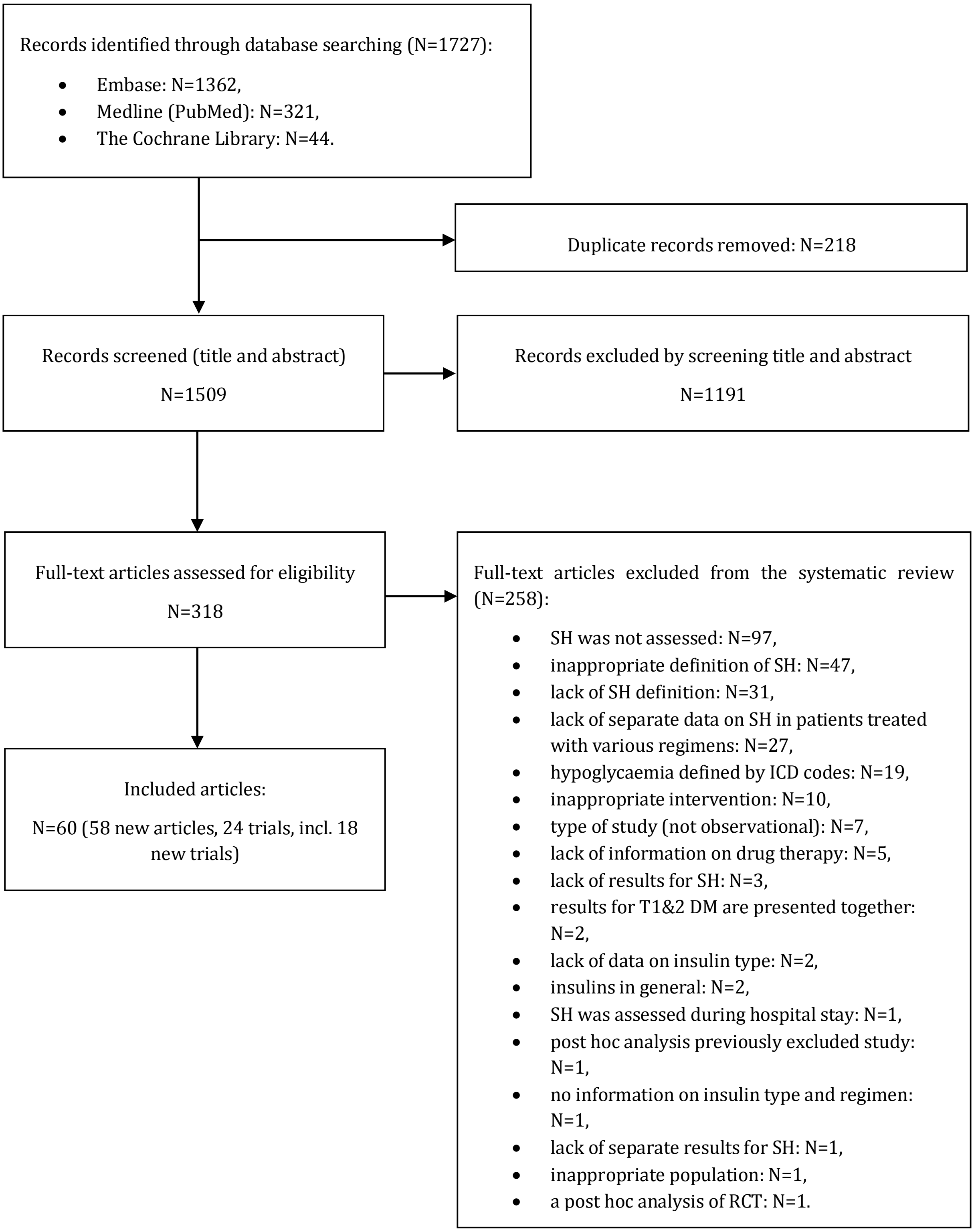

Figure 4. Supplementary systematic review of secondary studies (risk related to OADs in T2) – the study selection process.

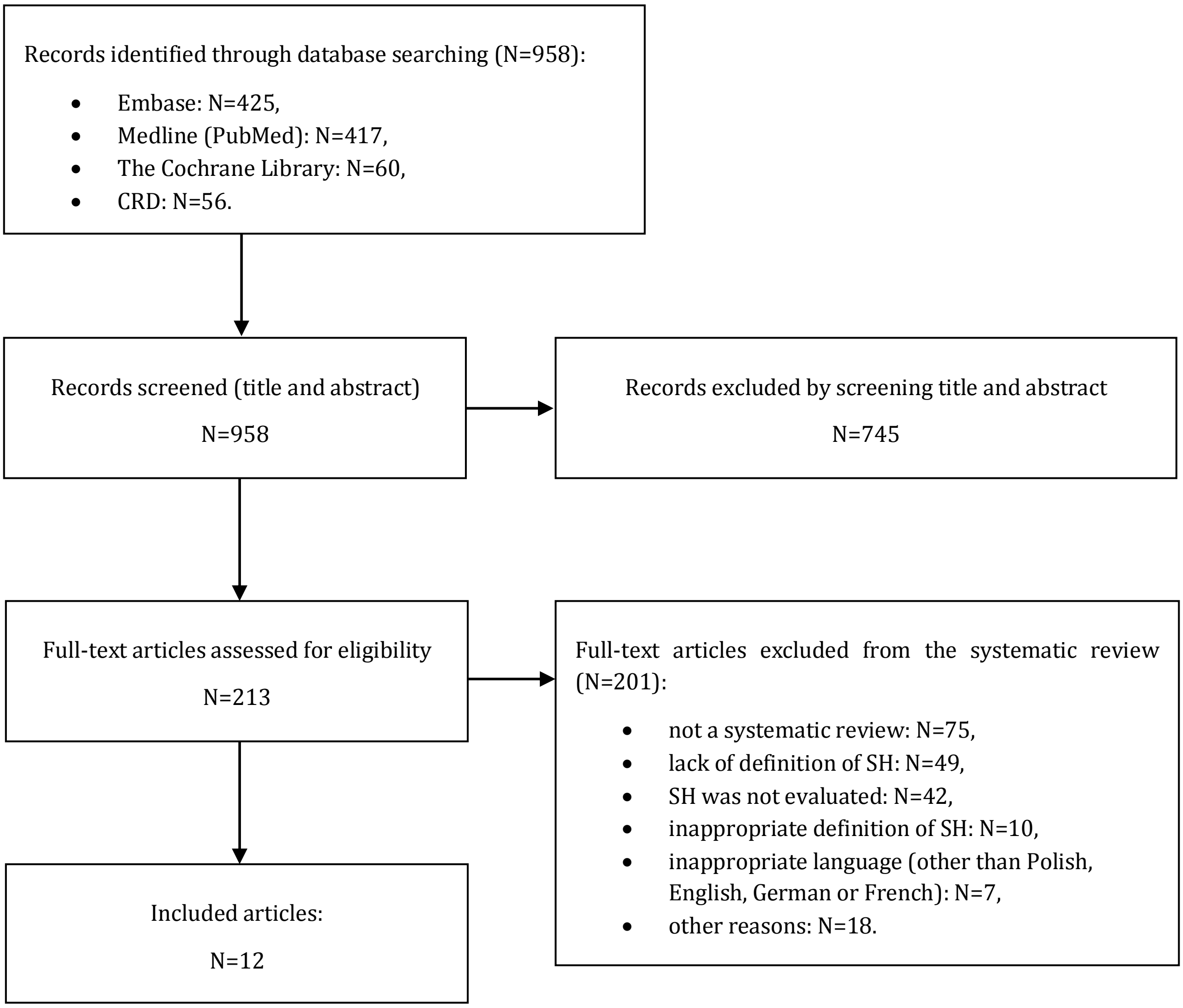

Figure 5. Supplementary systematic review of secondary studies (biphasic therapies in T1) – the study selection process.

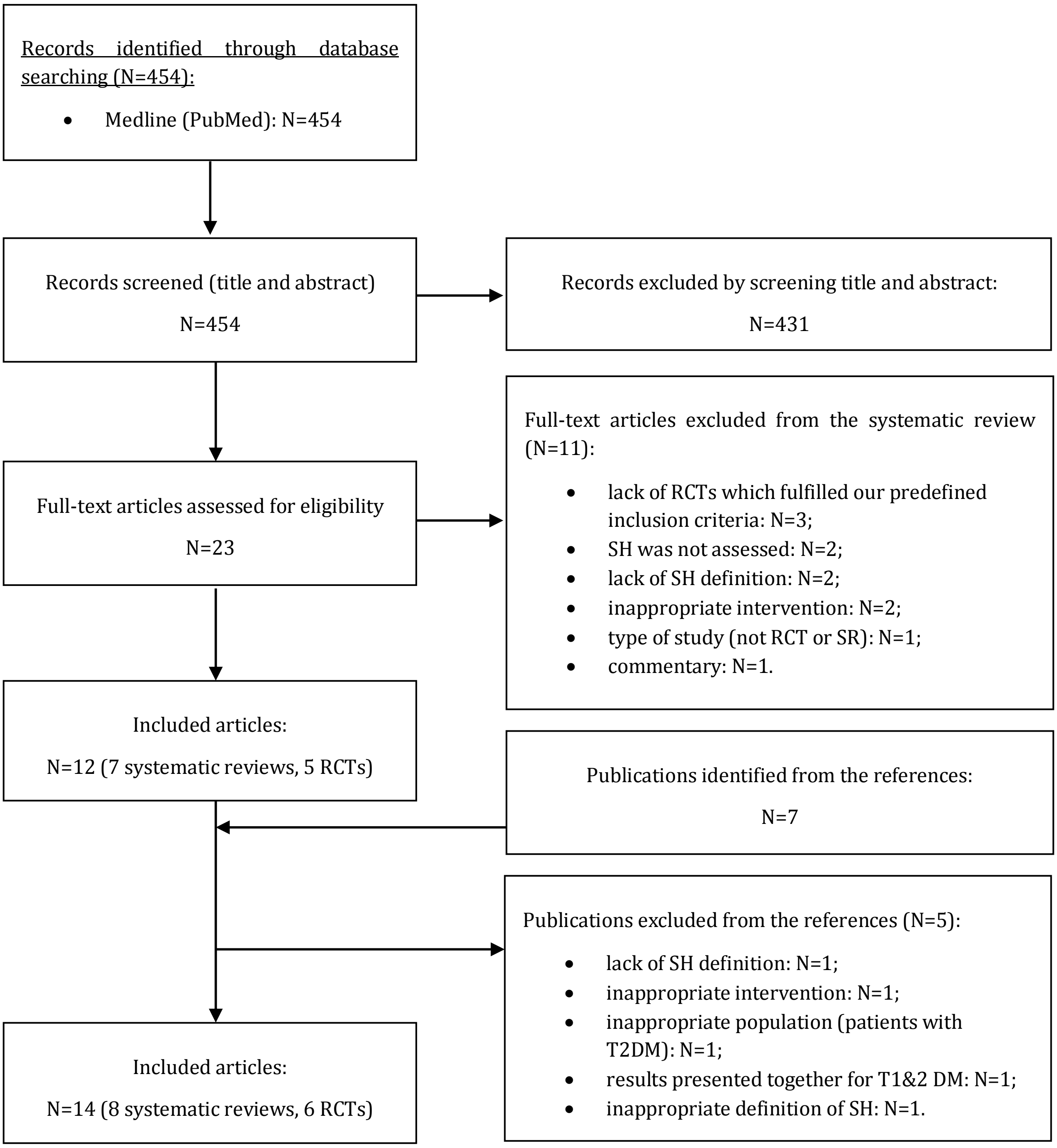

Supplement: Additional file 3: — Study selection process. (PDF 318 kb) [file 12902_2015_52_MOESM3_ESM.pdf]
