# Supplementary material for: Drug-related risk of severe hypoglycaemia in observational studies: a systematic review and meta-analysis
Source: BMC Endocr Disord. 2015 Oct 12;15:57. doi: 10.1186/s12902-015-0052-z (PMC4603823; doi:10.1186/s12902-015-0052-z)
Supplement: Additional file 4: — Studies characteristics. (PDF 332 kb) [file 12902_2015_52_MOESM4_ESM.pdf]

Characteristics of included studies for type 1 DM.

Study and country	Observation time, number of participants and design	Treatment of type I DM [a]	Patients characteristics [a]
Bruttomesso 2002<br>*Italy*	Mean 7.4 years<br>N= 138<br>Retrospective	Insulin pump	Adults, age at CSII start, (mean): 33.1 (11.74) years<br>Duration of diabetes, (mean): 13.1 (8.22) years<br>Patients treated with continuous subcutaneous insulin infusion for (mean):: 7.4 (4.70) years
DAFNE, Keen 2012<br>*UK*	1 year<br>N=124<br>Prospective	Basal long-acting insulin analogue + bolus insulin	Adults, mean age: 42.5 (11.1) years<br>Diabetes mean duration: 17.8 (11.0) years
de Bock 2012<br>*New Zeland*	3 years<br>N=621 (N=75 on CSII)<br>Retrospective	Insulin pump	Children, mean age at diagnosis: 6.0 (3.6) years<br>Previous treatment: patients had to use CSII for at least 12 months
Garg 2004a<br>*USA*	Mean 13.1 months<br>N=292<br>Retrospective	Basal long-acting insulin analogue + bolus insulin	Adult, mean age: 32 (10.0) years<br>Diabetes mean duration: 15.9 (10.3)<br>Previous treatment: at least 1 year of insulin therapy prior to beginning insulin glargine therapy and receiving intensive insulin therapy including at least four insulin injections per day with premeal short-acting insulin lispro or insulin aspart
Garg 2004b<br>*USA*	Follow-up period: from April 2001 to March 2003<br>13.1 months (basal bolus analogue group), N=98<br>12.7 months (basal bolus human group), N=98<br><br>Retrospective	Basal long-acting insulin analogue + bolus insulin<br><br>Human basal insulin + bolus insulin	Adults, mean age of patients in basal bolus analogue and human group, respectively: 33.4 (10.1) and 31.1 (8.7) years<br>Diabetes duration, mean: 16.0 (10.4) and 16.8 (9.2) years<br>Previous treatment with NPH in basal-bolus regimen

Study and country	Observation time, number of participants and design	Treatment of type I DM [a]	Patients characteristics [a]
Garg 2004c<br>*USA*	13.1 months, insulin glargine, N=299<br>11.6 months insulin pump, N=216<br><br>Retrospective	Basal long-acting insulin analogue + bolus insulin<br><br>Insulin pump	Adults, mean age: 32.0 (10.2) years<br>Diabetes duration, mean: 16.3 (9.9) years<br>Previous therapy: at least 1-year of insulin therapy followed by using of insulin pumps or insulin glargine; treatment with insulin glargine as their only basal insulin for at least 6 months
Hartemann-Heurtier 2003<br>*France*	1 year<br>N=110<br>Prospective	Human basal insulin + bolus insulin	Adults, mean age 45 (13) years<br>Diabetes duration, mean 17 (11) years<br>Previous therapy: regular human insulin before each meal (multiple injection/intensive therapy)
Hendrieckx 2014<br>*Australia*	6 months<br>N=422 (N=109 on insulin pump)<br>Retrospective	Insulin pump	Adults, mean age in total sample: 37.5 (SD=15.0)<br>Diabetes mean duration in total sample: 18.4 (SD=11.9)
Herwig 2007<br>*Germany*	1.68 years (mean)<br>N=142<br>Prospective	Basal long-acting insulin analogue + bolus insulin<br><br>Human basal insulin + bolus insulin	Children, age (range): 5-18 years<br>Duration of diabetes: >1 year
INTERPRET<br>*Austria, Belgium, Czech Republic, Denmark, France, Hungary, Israel, Italy, Lithuania, Poland, Serbia, Slovakia, Slovenia, Spain, Sweden*	12 months<br>N=274<br>Prospective	Insulin pump	Children and adults, mean age: 28.04 (SD=15.55) years<br>Diabetes mean duration: 13.87 (SD=10.67) years<br>Previous treatment: patients had to use CSII for at least 6 months
Jakisch 2008<br>*Germany, Austria*	3 years<br>N=828 (N=412 on insulin pumps)<br>Prospective, multicentre, matched pair cohort analysis	Insulin pump	Children, mean age: 10.9 years (median 11.4; iqr: 8.7-13.7)<br>Mean duration of diabetes: 3.5 years (median 2.7; iqr 1.3–5.2)<br>Previous treatment: conventional or multiple daily injections

Study and country	Observation time, number of participants and design	Treatment of type I DM [a]	Patients characteristics [a]
Kapellen 2007<br>*Germany, Austria*	33 months<br>N=1567<br>Retrospective	Insulin pump	Children and adolescents, mean age: 12.2 (4.2) years<br>Mean duration of diabetes: 5.2 (3.5) years
Kapellen 2009<br>*Germany, Austria*	12 years – 1-year assessment of SHEs after switching to insulin analogue (short- or long-acting)<br>N=37 206 (N=6558 on BB with long-acting insulin analogue)<br>Retrospective	Basal long-acting insulin analogue + bolus insulin	Children, mean age: 13.3 (4.4, all participants, not only from these 2 groups considered in the SR) years<br>Diabetes duration, mean: 5.2 (4.1, all participants) years<br>Patients had to be on insulin therapy for at least 2 years
Katz 2012<br>*USA*	0.9 year (basal-bolus group), N=50<br>1.57 year (CSII group), N=93<br>Prospective	Insulin pump<br><br>Basal long-acting insulin analogue + bolus insulin	Children: median 12.2 (range 9.0–15.0) years<br>Diabetes duration median 4.4 (range 1.0–13.0) years<br>To be included in the study patients had to be treated with: CSII; pump therapy, a basal insulin analogue (detemir or glargine) or NPH
Kristensen 2012<br>*Denmark*	1 year<br>N=3137 (N=1052 on basal insulin analogue and N=2085 on human insulin)<br>Retrospective	Basal long-acting insulin analogue + bolus insulin<br><br>Human basal insulin + bolus insulin	Adults, mean age: 48.0 (14.8) years<br>Diabetes duration, median 22 (range 0-73) years
Laubner 2014<br>*Austria, Germany*	Follow-up period: from January 2000 to September 2011<br>N=14 681<br>Retrospective	Basal long-acting insulin analogue + bolus insulin<br><br>Human basal insulin + bolus insulin	Adults, mean age: 37.7 (SD=19.8) years<br>Diabetes mean duration: 14.9 (SD=12.8) years<br>Previous treatment: insulin therapy
Leckie 2005<br>*Scotland*	1 year<br>N=243<br>Prospective	Human basal insulin + bolus insulin	Adults, range: from 20 to 69 years<br>Diabetes duration: n.a.<br>Previous therapy: n.a.

Study and country	Observation time, number of participants and design	Treatment of type I DM [a]	Patients characteristics [a]
Levy-Shraga 2013 *Israel*	Mean 9.6 (SD=7.0) years<br>N=113 (26 patients in considered group)<br>Retrospective	Insulin pump	Children, (Group 1 with CSII initiation before age 6 years) mean age at diagnosis: 2.4 (SD=1.3); mean age at CSII initiation: 3.4 (SD=1.4) years<br>Diabetes mean duration: 4.7 (SD=2.5) years
Leinung 2010 *USA*	1 year<br>N=117<br>Retrospective	Insulin pump	Adults, mean age 44.5 (12.8, range: 14.3-71.7, median: 46) years<br>Diabetes duration, mean 23.9 years<br>Patients were using insulin pumps before index date (i.e. initiation of CGM)
Maiorino 2014 *Italy*	12 weeks<br>N=125 (N=38 on insulin pump)<br>Prospective	Insulin pump	Young adults, mean age in CSII group: 21.7 (SD=2.3) years<br>Diabetes mean duration: 9.1 SD=4.0) years<br>Previous treatment: multiple daily injections
Maltoni 2013 *Italy*	Follow-up period: from January 1990 to December 2010 – 2212.9 patient-years (49.5 patient-years in insulin pump group) (N=36 on insulin pump)<br>N=269<br>Retrospective	Insulin pump	Children and young adults, mean age at T1DM onset: 7.6 (SD=4.1) years
Muller-Godeffroy 2009 *Germany*	0.5 year<br>N=114<br>Prospective	Insulin pump	Children, mean age: 10.5 (3.7) years<br>Diabetes duration, mean: 3.8 (2.9) years<br>Previous treatment: short-acting analogue, NPH, semilente or long-acting analogues
Nimri 2006 *Israel*	1 year<br>N=279<br>Retrospective	Insulin pump	Children, median age: 5.4 (range 1.6–8.6) years<br>Adolescents, median age: 13.7 (range 9–17) years<br>Adults, median age: 22.8 (range: 17–40) years<br>Previous therapy, multiple daily injections with mean duration: 8.3 (6.9) years<br>Duration of CSII: 2.4 (1.8) years
PREDICTIVE Marre 2009 *France*	1 year<br>N=1772 (N=643 with type 1 diabetes)<br>Prospective	Basal long-acting insulin analogue + bolus insulin	PREDICTIVE French cohort<br>Adults, mean age (SD): 47.1 (16.1) years<br>Diabetes mean duration (SD): 17.0 (12.0) years

Study and country	Observation time, number of participants and design	Treatment of type I DM [a]	Patients characteristics [a]
PREDICTIVE Preumont 2009<br>*Belgium*	0.5 year<br>N=232<br>Prospective	Basal long-acting insulin analogue + bolus insulin	PREDICTIVE Belgian cohort - only patients who were previously treated with NPH or glargine as basal insulin<br>Adults, mean age (SD): 45 (15) years<br>Diabetes mean duration (SD): 18 (13) years
PREDICTIVE Sreenan 2008<br>*Austria, Czech Republic, Denmark, Finland, Germany, Ireland, Israel, the Netherlands, Sweden, Turkey, UK*	12 weeks prospectively and 4 retrospectively<br>N=3637 (N=1500 with type 1)<br>Prospective with retrospective assessment	Basal long-acting insulin analogue + bolus insulin<br><br>Human basal insulin + bolus insulin	PREDICTIVE – only patients who were previously treated with NPH as basal insulin<br>[b] Adults, mean age (SD): 40.9 (17.2)<br>[b] Diabetes mean duration (SD): 14.1 (11.5)
PREDICTIVE Yenigun 2008<br>*Austria, Czech Republic, Denmark, Finland, Germany, Ireland, Israel, the Netherlands, Sweden, Turkey, UK*	12 weeks prospectively and 4 retrospectively<br>N=1285 (N=508 with type 1)<br>Prospective with retrospective assessment	Basal long-acting insulin analogue + bolus insulin	PREDICTIVE – only patients who were previously treated with glargine as basal insulin<br>Adults, mean age: 44.45 years<br>Diabetes mean duration: 18.85 years
Reda 2007<br>*New Zeland*	315 patient-years; mean 3 years<br>N=105<br>Retrospective	Insulin pump	Adults and children, mean age at CSII start: 33.1 (15.9; range 6.5-66.2) years<br>Diabetes duration, mean at CSII start: 18.2 (10.9; range 2-47) years<br>Previous therapy: patients had to be on CSII from at least 1 year, mean CSII duration: 3.0 (2.6); switched from MDI, mean duration: 7.2 (4.6)
Rudolph 2002<br>*USA*	36.1 months<br>N=107<br>Retrospective review of medical records	Insulin pump	Adults, mean age 36.0 (10.4) years<br>Diabetes duration at the CSII initiation 17.0 (9.1) years<br>Patients had to receive CSII therapy with more than 2 visits for clinical data collection

Study and country	Observation time, number of participants and design	Treatment of type I DM [a]	Patients characteristics [a]
Scaramuzza 2011<br>*Italy*	1.7 years, retrospective<br>1.4 years, prospective<br>N=622 (N=493 on insulin pump)<br>Multicenter, observational before-after study	Insulin pump	Children, mean age: 12.9 (3.4) years<br>Diabetes duration, mean: 6.2 (3.3) years<br>Children on pump therapy for mean 1.7 (0.5) years
Scheidegger 2007<br>*Switzeland*	104 patient-months<br>N=159<br>Prospectively	Insulin pump	Adults, mean age: 42.8 (11.2) years<br>Diabetes duration, mean: 18.2 (11.0) years<br>Patients previously treated with MDI
Wood 2006<br>*USA*	3.8 years of follow-up<br>N=161<br>Prospective	Insulin pump	Children and young adults, mean age: 14.1 (3.7; range 3.7-21.7), years<br>Diabetes duration, mean: 7.1 (4.0; range 0.7-16.7), years<br>Previous therapy: n.a.; patients had to start CSII therapy between 1 Jan 1998 and 31 Dec 2001
Ziegler 2013<br>*France, Germany, Italy, Spain, Sweden*	6 months<br>N=299<br>Prospective	Insulin pump	Adults, mean age: 39.4 (SD=15.2) years<br>CSII mean duration: 7.0 (SD=5.2) years<br>Previous treatment: patients had to use CSII for at least 6 months

[a] Data are presented only for patients groups treated according with scheme assumed in the SR and with type 1 DM unless no separate data on a group of interest was available (then characteristics of a whole study are shown). Means are presented with SD (if available).

Characteristics of included studies for type 2 DM.

Study and country	Observation time, number of participants and design	Treatment of type II DM [a]	Patients characteristics [a]
A_1chieve, Home 2011<br>*China; South Asia (Bangladesh, India, Pakistan); East Asia (Indonesia, Korea, Malaysia, Philippines, Singapore, Taiwan); north Africa (Algeria, Morocco, Tunisia, Libya); Middle East/Gulf (Egypt, Iran, Jordan, Turkey, Bahrain, Kuwait, Oman, Qatar, Saudi Arabia, UAE, Yemen); Latin America (Argentina, Mexico), Russia*	24 weeks<br>N=66 726 (N=40 909 on biphasic insulin analogue, N=15 545 on BOT with long-acting insulin analogue, N=4105 on BB with long-acting insulin analogue)<br>International, prospective, multicentre, non-interventional, observational study	Pre-mix insulin analogue ± OADs<br>Basal long-acting insulin analogue ±OADs<br>Basal bolus with basal long-acting insulin analogue ±OADs	Adult, mean age: 54.0 (12.0) years<br>Diabetes mean duration: 8.0 (6.2) years<br>Previous treatment: prior to enrolment in the study, 58.2% of patients were being treated with OADs alone, 23.8% were receiving OADs + insulin therapy, 8.1% insulin only and 9.0% no medication for diabetes
A_1chieve, El-Naggar 2012<br>*China, South Asia, East Asia, North Africa, Middle East/Gulf*	24 weeks<br>N=6323<br>Prospective	Pre-mix insulin human<br>Pre-mix insulin analogue	Adults, mean age: 55.4 (12.5) years<br>Diabetes mean duration: 11.1 (6.6) years
A_1chieve, El-Naggar 2013<br>*Gulf*	24 weeks<br>N=10 704 (N=6153 in insulin-naive group and N=4551 in prior insulin users group)<br>Prospective	Pre-mix analogue	Adults, mean age of insulin-naïve and prior insulin users group, respectively: 50.4 (9.7) and 51.5 (12.6) years<br>Diabetes mean duration of insulin-naïve and prior insulin users group, respectively: 8.5 (5.1) and 11.7 (6.5) years
Andayani 2010<br>*Indonesia*	6 month<br>N=115 (N=49 received SU)<br>Prospective	Sulfonylurea	Adult, mean age: 62.84 (7.85) years<br>Diabetes duration, mean 11.92 (6.09) years<br>Patients failing with oral antidiabetic medication
Aung 2012<br>*Scotland*	1 year<br>N=1066<br>Cross-sectional, population-based study	Sulfonylurea	Adult, mean age: 67.9 (4.2) years<br>Diabetes mean duration: 8.1 (6.5) years

BIAsp Start, Berntrop 2011<br>*Sweden*	27 weeks<br>N=1154<br>Prospective, observational, multicentre study	Pre-mix insulin analogue ±OADs	Adult, mean age: 65 (11.5) years<br>Diabetes mean duration: 8.1 (6.2) years<br>Previous treatment: patients with or without previous OAD treatment
Biesenbach 2006<br>*Austria*	1 year<br>N=154 (N=34 on BB with NPH)<br>Retrospective	Basal bolus with NPH ±OADs	Adult, mean age: insulin only: 67.7 (11.9) and 68.5 (9.2); SU + insulin 73.7 (8.9) years<br>Diabetes duration, mean: insulin only 9 (range 3-12) and 8 (2-9); SU + insulin 7 (2-8) years<br>Patients newly treated with insulin due to failure of secondary SU treatment
Buturovic 2013<br>*Bosnia and Herzegovina*	9 months<br>N=278<br>Prospective	Basal bolus with basal long-acting insulin analogue	Adults, mean age: 60.2 (SD=8.8) years<br>Diabetes mean duration: 10.1 (SD=6.4) years<br>Previous treatment: at least 2 daily injections of pre-mixed insulin for at least 6 months
Danish BIAsp Study Group, Breum 2008<br>*Denmark*	26 weeks<br>N=421<br>Prospective	Pre-mix insulin analogue ± OADs	Adult, mean age: 62.0 (11.4) years<br>Diabetes mean duration: 9.1 (8.1) years<br>Previous treatment: 199 subjects were prior insulin users and 193 were insulin naïve patients
EARLY, Hanefeld 2012<br>*Germany*	24 weeks<br>N=1438 (N=1389 on BOT with long-acting insulin analogue)<br>Prospective	BOT with long-acting insulin analogue	Adult, mean age: 65.56 (10.68) years<br>Diabetes duration, mean: 7.06 (5.45) years<br>Patients treated with maximally tolerated metformin doses for at least three months
Echtay 2013<br>*Israel, Jordan, Pakistan, Lebanon*	24 weeks<br>N=2155<br>Prospective	BOT with long-acting insulin analogue	Adults, mean age: 57.2 (SD=11.0) years<br>Diabetes mean duration: 9.2 (SD=5.4) years<br>Previous treatment: at least one oral anti-diabetic drugs (metformin, sulfonylureas, repaglinide, thiazolidinediones)

Exhype, Pettersson 2011<br>*Sweden*	6 months<br>N=430<br>Retrospective	Sulfonylurea	Adult, mean age: 69.0 (9.5) years<br>Diabetes duration >7 years: 71% of patients<br>Participants had to be treated with metformin and SU for at least 6 months (insulin naive)
FINE, Tsai 2011<br>*Bangladesh,China, Hong Kong, India, Indonesia, Korea, Pakistan, Singapore, Taiwan, Thailand, Vietnam*	6 months<br>N=2679 (N=2077 on BOT with long acting-insulin analogue and N=589 on BOT with NPH)<br>Multinational, prospective, observational study	Basal long-acting insulin analogue ±OADs<br>Basal NPH ±OADs	Adult, mean age: 56.4 (11.2) years<br>Diabetes mean duration: 9.3 (6.5) years
Furlong 2002<br>*UK*	29 months (median)<br>N=208<br>Retrospective	BOT with NPH	Adult, mean age: metformin 58.6 (9.0), SU 64.3 (9.6) years<br>Diabetes duration: metformin 7 (1-25), SU 9 (1-29) years<br>Patients inadequately controlled with OADs
Gomez-Peralta 2012<br>*Spain*	6 months<br>N=131<br>Retrospective	BOT long-acting analogue	Adults, mean age: 68.2 (9.4) years<br>Diabetes mean duration: 12.7 (6.9) years
Gu 2012<br>*China*	16 weeks<br>N=660<br>Prospective, open-label, multicentre, observational	Pre-mix human insulin ± OADs	Adult, median age: 61.9 (iq: 52.7, 71.4) years<br>Diabetes duration, median: 6.7 (ig: 3.7, 10.2) years<br>Patients treated with ≥2 OADs (≥1 insulin secretagogue) and insulin-naive
Guo 2013<br>*China*	16 weeks<br>N=391<br>Prospective	Sulfonylurea	Adults, mean age: 53.1 (SD=10.3) years<br>Diabetes mean duration: 22.5 (SD=38.8) months<br>Previous treatment: none

Honkasalo 2010, Honkasalo 2011<br>*Finland*	1 year<br>N=680 (N=431 with type 2 diabetes)<br>Retrospective	Basal NPH ± OADs	Adult, median age: 53.1 (8.6) years<br>Diabetes duration: n.a.
IMPROVE, Gumprecht 2009<br>*Canada, China, Greece, Gulf region, India, Iran, Italy, Japan, Poland, Russia, South Korea*	13 weeks<br>N=748 (N=497 on prestudy BOT with NPH and N=245 on prestudy BOT with long acting-insulin analogue)<br>Only results of retrospective assessment are included in the SR	Basal long-acting insulin analogue ±OADs (prestudy)<br>Basal NPH ± OADs (prestudy)	IMPROVE – only patients who were previously treated with basal insulin<br>Mean age (SD): 59.7 (11.8) years<br>Mean diabetes duration (SD): 11.4 (7.3) years
IMPROVE, Khader 2010<br>*Saudi Arabia, United Arab Emirates, Kuwait, Qatar and Oman*	6 months<br>N=1613<br>Prospective	Pre-mix insulin analogue ±OADs	IMPROVE - Gulf region, all patients<br>Mean age (SD): 50.5 (10.4) years<br>Mean diabetes duration (SD): 10.1 (5.8) years
IMPROVE, Shah 2009 a<br>*Canada, China, Greece, Gulf region, India, Iran, Italy, Japan, Poland, Russia, South Korea*	13 weeks<br>N=3856<br>Only results of retrospective assessment are included in the SR	Pre-mix human insulin ±OADs	IMPROVE – only patients who were previously treated with biphasic human insulin 30 ±OADs<br>Mean age (SD): 57.0 (11.5) years<br>Mean diabetes duration (SD): 10.7 (6.9) years
IMPROVE, Valensi 2009<br>*Canada, China, India, Japan, Poland, Russia, Greece, Italy*	6 months<br>N=52 419<br>Prospective	Pre-mix insulin analogue ±OADs	IMPROVE – 8 countries, all patients<br>Mean age (SD): 55.3 (11.9) years<br>Mean diabetes duration (SD): 7.0 (6.1) years
INITIATEplus, Oyer 2011<br>*USA*	24 weeks<br>N=4875<br>Multicenter, open-label, prospective study	Pre-mix insulin analogue ±OADs	Adult, mean age: 54.0 (2.01) years<br>Diabetes mean duration: n.a.<br>Previous treatment: n.a.

Ivanyi 2012<br>*Hungary*	132 weeks<br>N=155<br>Prospective	Sulfonylurea	Adult, range: 20-75, mean age 58.7 (9.0) years<br>Diabetes duration, mean: 9.1 (5.9) years<br>Patients treated with metformin, SU or both
JDDM23, Oishi 2012<br>*Japan*	6 months<br>N=153<br>Prospective with 12-week retrospective assessment	Basal bolus with NPH ± OADs<br>Basal bolus with long-acting insulin analogue ± OADs	Adult, mean age: 60.2 (10.8) years<br>Diabetes mean duration: 13.6 (9.2) years<br>Previous treatment: NPH insulin
Kawamori 2008<br>*Brazil (Japanese patients)*	0.46 year<br>N=100<br>Prospective	BOT with long-acting insulin analogue	Adult, mean age: 58.4 (7.7; median 59.0) years<br>Diabetes duration, mean: 11.5 (6.6) years<br>Patients inadequately controlled with SU (± other OAD); glimepiride was used for at least 2 months before study
Klen 2014<br>*Slovenia*	3 months<br>N=156<br>Prospective	Sulfonylurea	Adults, mean age: 63.6 (SD=7.7) years<br>Diabetes mean duration: 10.6 (SD=7.1) years<br>Previous treatment: sulphonylurea in monotherapy or in combination with metformin for at least six months
Kulzer 2014<br>*Germany*	1771 respondent-weeks<br>N=614 (N=91 with T2DM on long-acting insulin only; N=253 with T2DM on short- and long-acting insulin)<br>Retrospective	Basal long-acting insulin analogue<br><br>Basal bolus with basal long-acting insulin analogue	Adults, mean age in T2DM group: 53.10 (SD=12.23) years<br>Diabetes average duration in T2DM group: 11.6 (SD=8.6) years<br>Previous treatment: insulin
Laubner 2014<br>*Austria, Germany*	Follow-up period: from January 2000 to September 2011<br>N=37 283<br>Retrospective	Basal bolus with basal long-acting insulin analogue<br><br>Basal bolus with NPH	Adults, mean age: 71.2 (SD=11.3) years<br>Diabetes mean duration: 14.6 (SD=9.7) years<br>Previous treatment: insulin therapy

Levit 2011<br>*Israel*	Mean (SD) 2.9 year (0.9)<br>N=115<br>Retrospective	Pre-mix insulin analogue ± OADs	Adult, mean age: 62.4 (12.2) years<br>Diabetes duration: 10.1 (8.0) years<br>Prior therapy: one or more OADs or insulin therapy (basal, prandial, basal-bolus)
LIGHT, Verges 2012<br>*France*	3 months<br>N=2541<br>Longitudinal, observational study	BOT with long-acting insulin analogue	Adult, mean age: 63.7 (10.1) years<br>Diabetes mean duration: n.a.
Ligthelm 2009<br>*Denmark*	18 months<br>N=150<br>Prospective	Pre-mix insulin analogue ± OADs	Adult, mean age of insulin naïve group, NPH group, insulin glargine group and BHI 30 group, respectively: 56.7 (range 34–79), 59.4 (range 41–77), 59.4 (range 41–77) and 60.7 (range 51–78) years<br>Diabetes mean duration of insulin naïve group, NPH group, insulin glargine group and BHI 30 group, respectively: 6.7 (range 4.2–9.7), 8.3 (range 6.5–14.3), 7.8 (range 4.4–9.6) and 10.9 (range 7.1–18.2) years
Makela 2012<br>*Finland*	26 weeks<br>N=557<br>Observational, non-interventional, non-randomised, open-label, prospective	Pre-mix insulin analogue ± OADs	Adult, mean age: 65.8 (range 30.0–90.0) years<br>Diabetes mean duration: 11.4 (6.9) years
Nakashima 2013<br>*Japan*	48 weeks (3x16 weeks)<br>N=135<br>Prospective	Pre-mix insulin analogue	Adults, mean age: 60.3 (SD=10.2) years<br>Diabetes mean duration: 11.4 (SD=7.1) years<br>Previous treatment: OADs for at least 90 days prior to the start of the study and no insulin therapy within 6 months prior to the study

Nobels 2012<br>*Belgium, Luxembourg*	26 weeks<br>N=592<br>Prospective with 4-week prestudy data	Pre-mix insulin analogue ± OADs<br>Pre-mix human insulin ± OADs	Adult, mean age: 68.0 (10.6) years<br>Diabetes mean duration: 14.6 (8.9) years<br>Previous treatment: BHI therapy
Obstacle Hypoglycaemia Study, Kalra 2013<br>*India*	12 weeks<br>N=1069<br>Prospective	Sulfonylurea	Adults, mean age: 52.21 (SD=10.70) years<br>Diabetes mean duration: 3.80 (SD=3.82) years<br>Previous treatment: OADs
Ostenson 2014<br>*Austria, Denmark, Finland, Norway, Sweden, Switzer-land, the Netherlands*	11 440 respondent-weeks<br>N=3827 (N=812 with T2DM on long-acting insulin only; N=942 with T2DM on short- and long-acting insulin)<br>Retrospective	Basal long-acting insulin analogue<br><br>Basal bolus with basal long-acting insulin analogue	Adults, mean age in T2DM group: 60.3 (SD=10.7) years<br>Diabetes mean duration in T2DM group: N=41 (<2 years); N=317 (2-5 years); N=394 (5-9 years); N=546 (10-14 years); N=806 (15+ years)<br>Previous treatment: insulin
Panelo 2013<br>*Philippines*	6 months<br>N=2370<br>Prospective	Sulfonylurea	Adults, mean age: 54.32 (SD=11.56) years<br>Diabetes mean duration: 5.16 (SD=4.72) years<br>Previous treatment: oral drug therapy
Pirags 2012<br>*China, Croatia, Czech Republic, Egypt, Estonia, Latvia, Poland, Romania, , Slovak Republic, Turkey*	12 months<br>N=1139<br>Prospective	Pre-mix insulin analogue	Adults, mean age: 57.9 (10.1) years<br>Diabetes mean duration: 9.2 (5.9) years
PREDICTIVE, Dornhorst 2008 b<br>*Austria, Czech Republic, Denmark, Finland, Germany, Ireland, Israel, the Netherlands, Sweden, Turkey, the UK*	4-week retrospectively<br>N=293 (N=175 on BOT with NPH and N=118 on BOT with long-acting insulin analogue)<br>Only results of retrospective assessment are included in the SR	BOT with long-acting insulin analogue<br>BOT with NPH	PREDICTIVE – patients who were previously treated with BOT<br>Adult, mean (SD) age: 60.9 (10.9) years and 63.5 (11.4), NPH and glargine group respectively<br>Diabetes mean (SD) duration: 11.6 (7.0) years and 9.9 (6.5), NPH and glargine group respectively
PREDICTIVE, Meneghini 2009<br>*Denmark, Finland, Sweden, Austria, Germany, The Czech Republic, Israel, Turkey, the Netherlands, the United Kingdom, Ireland*	12 weeks<br>N=1832<br>Prospective	BOT with long-acting insulin analogue	PREDICTIVE – sub-analysis of cohort of insulin-naïve patients<br>Mean (SD) age: 60.8 (10.9) years<br>Mean (SD) diabetes duration: 8.0 (5.9) years

PREDICTIVE, Sreenan 2008	12 weeks<br>N=3637 (N=2137 with type 2 diabetes)<br>Prospective with 4-week retrospective assessment	Basal-bolus with long-acting insulin analogue<br>Basal-bolus with NPH	PREDICTIVE – patients who were previously treated with NPH + bolus<br>[b] Mean (SD) age: 60.5 (10.2) years<br>[b] Mean (SD) diabetes duration: 12.7 (7.3) years
PRESENT, Gao 2009<br>*China*	12 week<br>N=11 662<br>Prospective	Pre-mix insulin analogue	PRESENT – total China cohort<br>Mean (SD) age: 50.0 (11.2), 54.6 (10.9), 55.7 (11.7), 57.8 (10.9) years, patients who were drug-naïve, on OADs only, on inslulin only and treated with insulin +OADs, respectively<br>Mean (SD) diabetes duration: 2.9 (3.7), 5.5 (4.2), 6.4 (4.9), 7.3 (4.9) patients split by prestudy therapy: drug-naïve, OAD, insulin, insulin+OAD, respectively
PRESENT, Jang 2008<br>*India, Iraq, Jordan, Kuwait, Lebanon, Qatar, Romania, Russia, Saudi Arabia, South Africa, South, Korea, Turkey, the United Arab Emirates, China, Sri Lanka*	12-week<br>N=3762 (N=3414 on BOT with human insulin and N=348 on BOT with long-acting insulin analogue)<br>Retrospective assessment	Basal long-acting insulin analogue ±OADs<br>Basal NPH ±OADs	PRESENT - patients who were previously treated with basal insulin ±OADs<br>Mean (SD) age: 56.9 (12.0), 56.8 (12.2) years, patients treated previously with analogue basal insulin and human basal insulin, respectively<br>Mean (SD) diabetes duration: 9.9 (7.2), 10.9 (7.0) years, patients treated previously with analogue basal insulin and human basal insulin, respectively
PRESENT, Khutsoane 2008<br>*India, Iraq, Jordan, Kuwait, Lebanon, Qatar, Romania, Russia, Saudi Arabia, South Africa, South, Korea, Turkey, the United Arab Emirates*	26 weeks<br>N=21 977<br>Prospective	Pre-mix insulin analogue	PRESENT – total study cohort<br>Mean (SD) age: 55.3 (12.2) years<br>Mean (SD) diabetes duration: 9.7 (6.8) years

PRESENT, Shestakova 2007<br>*India, Iraq, Jordan, Kuwait, Lebanon, Qatar, Romania, Russia, Saudi Arabia, South Africa, South, Korea, Turkey, the United Arab Emirates*	12-week<br>N=3985<br>Retrospective assessment	Pre-mix human insulin	PRESENT - patients who were previously treated with pre-mix human insulin<br>Mean (SD) age: 55.67 (12.61) years<br>Diabetes duration, mean (SD): 11.10 (6.88) years
Progens-first step, Strojek 2008<br>*Poland*	2 periods each of 13-week duration<br>N=1384<br>Prospective, multicentre observational, conducted in the outpatient setting	Biphasic human insulin (Gensulin)	Adult, mean age: 61 (9.1) years<br>Diabetes duration, mean: 63 (54) months<br>Patients with secondary failure to OADs
RESOLUTE<br>*Czech Republic, Netherlands, Poland, Russia, Argentina*	6 months,<br>N=564<br>Prospective	BOT with long-acting insulin analogue	Adults, mean age: 61.9 (SD=10.1) years<br>Diabetes mean duration: 9.2 (SD=5.9) years<br>Previous treatment: insulin detemir ± OADs
SAFIR, Zick 2007<br>*Germany*	8 weeks<br>N=479<br>Multicentre, open-label, single-arm	Basal-bolus with long acting insulin-analogue ± OADs	Adult, mean age: 59.2 (8.2) years<br>Diabetes duration, mean 12.7 (7.4) years<br>Prestudy therapy: NPH with mealtime insulin for at least 3 months
SOLVE, Damci 2014<br>*Turkey*	24 weeks<br>N=2886 (N=491 in IGlar group)<br>Prospective	BOT long-acting insulin analogue	Adults, mean age in IGlar group: 56.6 (SD=10.3) years<br>Diabetes mean duration in IGlar group: 8.6 (SD=5.4) years<br>Previous treatment: oral anti-diabetic drugs
SOLVE, Khunti 2012<br>*Canada, China, Germany, Israel, Italy, Poland, Portugal, Spain, Turkey, UK*	24 weeks<br>N=17 374<br>Prospective	BOT long-acting analogue	Adults, mean age: 62 (12) years<br>Diabetes mean duration: 10 (7) years
Sudhakaran 2010<br>*India*	24 weeks<br>N=124 (N=23 on BOT with NPH and N=54 on BOT with long-acting insulin analogue)<br>Retrospective	Basal long-acting insulin analogue ± OADs<br>Basal NPH ±OADs	Adult, mean age of insulin glargine group and NPH insulin group, respectively: 54.6 (11.1) and 53.8 (8.1) years<br>Diabetes duration, mean of insulin glargine group and NPH insulin group, respectively: 12.9 (3.9) and 10.2 (4.6) years

Sudhakaran 2011<br>*India*	24 weeks<br>N=5560 (N=2743 on BOT with long-acting insulin analogue)<br>Retrospective	BOT with long-acting insulin analogue	Adult, mean age in insulin glargine group: 54.2 (9.2) years<br>Diabetes duration, mean in insulin glargine group: 13.2 (6.7) years
Suzuki 2012<br>*Japan*	1 year<br>N=400<br>Prospective	Basal bolus with long-acting insulin analogue	Adult, mean age: 65.6 (15.2) years<br>Previous therapy: NPH insulin and regular or rapidly acting bolus insulin for more than three months
Temizel 2010<br>*Turkey*	1 year<br>N=140 (N=71 on pre-mix insulin analogue and N=69 on pre-mix human insulin)<br>Retrospective	Pre-mix insulin analogue<br>Pre-mix human insulin	Adult, mean age: 60.42 (10.86) and 60.29 (9.04) years, human analogue group and regular insulin, respectively<br>Diabetes duration at insulin start, mean: 9.82 (7.14) and 9.75 (5.62) years, human analogue group and regular insulin, respectively
Tentolouris 2013,<br>*Greece*	6-12 months<br>N=301 (N=142 in IGlar group; N=159 in BHI group)<br>Retrospective	Basal long-acting insulin analogue ± OADs<br><br>Pre-mix human ± OADs	Adults, mean age in IGlar and BHI group, respectively: 63.2 (SD=10.1) years and 66.7 (SD=9.6) years<br>Diabetes mean duration: 13.5 (SD=7.8) years and 16.0 (SD=8.6) years<br>Previous treatment: biphasic human insulin twice daily in combination with OAD

The 1-2-3 study, Garber 2006<br>*USA*	16 weeks<br>N=193<br>Open-label, observational study	Pre-mix insulin analogue	Adult, mean age of once-daily Phase 1 group, twice-daily Phase 2 group and thrice-daily Phase 3 group, respectively: 56.7 (11.5), 58.0 (11.2) and 57.7 (10.2)<br>Diabetes mean duration of once-daily Phase 1 group, twice-daily Phase 2 group and thrice-daily Phase 3 group, respectively: 11.1 (7.1), 12.0 (7.3) and 12.1 (7.8)<br>Previous treatment: treatment with two or more OADs or treatment with one OAD and a<br>basal insulin (insulin glargine or NPH insulin, no more than 60 U/day)
UK Hypoglycaemia Study Group<br>*UK*	Mean 0.73 year<br>N=383 (N=108 received SU)<br>Prospective	Sulfonylurea	Adult, mean age: 60.8 (9.3) years<br>Diabetes duration, mean: 6.0 (3.6-9.7) years
Vexiau 2008<br>*France*	6 months<br>N=400<br>Observational, cross-sectional, multi-centre study	Sulfonylurea	Adult, mean age: 62.1 (10.7) years<br>Patients treated with SU and metformin for at least 6 months, insulin-naive
Yang 2012<br>*China*	16 weeks<br>N=313<br>Multicentre, single-arm, open-label study	BOT with long-acting insulin analogue	Adult, mean age: 56.97 (8.31) years<br>Diabetes mean duration: 7.46 (2.56) years<br>Previous treatment: pre-mix insulin plus OADs
Zjačić-Rotkvić 2012<br>*Croatia*	26 weeks<br>N=220<br>Prospective, open-label, single-arm, multicentre, postmarketing	Basal bolus with long-acting insulin analogue	Adult, mean age: 62.2 (9.2) years<br>Diabetes duration: 13.9 (7.6) years<br>Previous therapy: inadequate glycaemic control with pre-mix insulin

[a] Only groups included in the review are described unless data were provided only for a total study cohort. Means are presented with SD (if available).
