# Supplementary material for: Drug-related risk of severe hypoglycaemia in observational studies: a systematic review and meta-analysis
Source: BMC Endocr Disord. 2015 Oct 12;15:57. doi: 10.1186/s12902-015-0052-z (PMC4603823; doi:10.1186/s12902-015-0052-z)
Supplement: Additional file 5: — Individual studies data. (PDF 466 kb) [file 12902_2015_52_MOESM5_ESM.pdf]

Results from the included observational studies.

Table 1. Diabetes type 1 results of the included studies

Study	Time horizon (years)	Number of participants	Patients with ≥1 SHE	Number of events	Mean events no per patient-year	SD
**Insulin pump therapy**						
Bruttomesso 2002	7.4	138		92	0.09	0.02
de Bock 2012	3	75		11	5	
Garg 2004c	0.97	216	45	84	0.4	Not clear
Hendrieckx 2014	0.5	109	16			
INTERPRET	1	263	9	13	0.063	
Jakisch 2008	1	412; 300; 199		74; 60; 34	17.87; 20.04; 17.33	2.85; 3.91; 4.47
Kapellen 2007	1; 1; 2.75; 2.75	248; 544; 76; 177			0.25; 0.14; 0.27; 0.27	
Katz 2012	1.69	93		50	31.8	
Leinung 2010	1	117	37	68	58.9	
Levy-Shraga 2013	9.7	26			0.083	
Maiorino 2014	0.23	38	0	0		
Maltoni 2013	0.73	36			0.1	
Muller-Godeffroy 2009	0.5	88	6			
Nimri 2006	1; 1; 1	127; 129; 23			11.1; 23.3; 0	
Reda 2007	2.6	105		15	0.05	
Rudolph 2002	3.01	107			19.2	
Scaramuzza 2011	1.7; 1.4	493; 493			6.6; 3.9	
Scheidegger 2007	0.46	19	1	1		
Wood 2006	1	132			7.4	
Ziegler 2013	0.5; 0.25	299; 299	1	<0.4%*; <1%*		
**Basal bolus with long-acting insulin analogue**						
DAFNE, Keen 2012	1; 1	124; 124	15; 6	37; 22		
Garg 2004 a	1.09	292 (98, 299)	81 (28, 81)	167 (n.a., n.a.)	0.57 (0.5, 0.6)	
Herwig 2007	1.68	74		11	0.14	0.4
Kapellen 2009	1	6558			32.2/100	3
Katz 2012	1.8	50		31	34.4	
Kristensen 2012	1	1052			1.47	SE=0.18
Laubner 2014	11.75	5576; 2918	10.86%; 11.93%			
PREDICTIVE, Marre 2009	1	647		11	0.02	

Study	Time horizon (years)	Number of participants	Patients with ≥1 SHE	Number of events	Mean events no per patient-year	SD
PREDICTIVE, Preumont 2009	0.5	232			0.1	0.7
PREDICTIVE, Sreenan 2008	0.23	1500			0.52	
PREDICTIVE, Yenigun 2009	0.08; 0.23	506; 506		94; 28		
**Basal bolus with human basal insulin**						
Garg 2004b	1.06	98	30		1.2	SEM=0.40
Hartemann-Heurtier 2003	1; 1	110; 110	14; 26		0.2; 0.83	0.62; 3.34
Herwig 2007	1.68	68		62	0.73	1.68
Kristensen 2012	1	2085			1.09	SE=0.11
Leckie 2005	1	243	83		0.98	
Laubner 2014	11.75	6187	11.06%			
PREDICTIVE, Sreenan 2008	0.077	1500			3.51	

* we assumed value of 0.4% and 1%, respectively.

Table 2. Diabetes type 2 results of the included studies

Study	Time horizon (years)	Number of participants	Patients with ≥1 SHE	No of events – absolute or mean per patient-year
**Basal long-acting insulin analogue ± OADs**				
A1chieve, Home 2011	0.08	12 078 and 3467		0 and 0.01
EARLY, Hanefeld 2012	0.46	1389	1	1
Echtay 2013	0.08	2106		0.02
FINE, Tsai 2011	0.50	2016 and 61		0.003 and 0
Gomez-Peralta 2012	0.50	131	2	
IMPROVE, Gumprecht 2009	0.25	245		0.197
Kawamori 2008	0.46	97	0	0
Kulzer 2014	1	91		0.2
LIGHT, Verges 2012	0.25	1863	18	0.12
Ostenson 2014	1	812		0.1
PREDICTIVE, Dornhorst 2008 b	0.08	118		0.26
PREDICTIVE, Meneghini 2009	0.23	1652		0.00
PRESENT, Jang 2008	0.23	348		1.1

Study	Time horizon (years)	Number of participants	Patients with ≥1 SHE	No of events – absolute or mean per patient-year
RESOLUTE	0.08	511 and 564	0% and 1.2%	0 and n.a.
SOLVE, Damci 2014	0.23	491	0	0
SOLVE, Khunti 2012	0.46	17 374	21	31 episodes; 0.05
Sudhakaran 2010	0.46	54	0	0
Sudhakaran 2011	0.46	2743	0	0
Tentolouris 2013	0.75	142		0.007 episodes/month
Yang 2012	0.31	297	2	2
**Basal human insulin ± OADs**				
FINE, Tsai 2011	0.50	589		0.031
Furlong 2002	2.42 (median)	133 and 67	6 and 1	
Honkasalo 2010, Honkasalo 2011	1	431	53 (12.3%)	116
IMPROVE, Gumprecht 2009	0.25	497		0.153
Laubner 2014	11.75	28 300	5.66%	
PREDICTIVE, Dornhorst 2008 b	0.08	175		0.78
PRESENT, Jang 2008	0.23	3414		0.39

Study	Time horizon (years)	Number of participants	Patients with ≥1 SHE	No of events – absolute or mean per patient-year
Sudhakaran 2010	0.46	23	0	0
**Basal bolus with long-acting insulin analogue ± OADs**				
A1chieve, Home 2011	0.08	1593 and 2512		0 and 0.001
Buturovic 2013	0.75	258	0	0
JDDM23, Oishi 2012	0.50	126	1	1
Kulzer 2014	1	253		0.5
Laubner 2014	11.75	6498 and 2485	5.03% and 4.48%	
Ostenson 2014	1	942		0.2
PREDICTIVE, Sreenan 2008	0.23	2137		0
SAFIR, Zick 2007	0.15	455	0.7%	0.05
Suzuki 2012	1	400	1	1
Zjačić-Rotkvić 2012	0.5	203	0	0
**Basal bolus with human insulin ± OADs**				
Biesenbach 2006	1	34		0.05 per patient-month
JDDM23, Oishi 2012	0.23	126	1	1
PREDICTIVE, Sreenan 2008	0.08	2137		0.78 per patient year

Study	Time horizon (years)	Number of participants	Patients with ≥1 SHE	No of events – absolute or mean per patient-year
**Pre-mix insulin analogues**				
A_1chieve, El-Naggar 2012	0.08	6323	4	12 events; 0.03 events/patient-year
A_1chieve, El-Naggar 2013	0.08	6153 and 4551	0.05% and 0.1%	0.01 and 0.01
A_1chieve, Home 2011	0.08	27 591 and 13 318		0 and 0.02 per patient-year
BIAsp Start, Berntorp 2011	0.52	1154	2	2
Danish BIAsp Study Group, Breum 2008	0.5	392	4	
IMPROVE, Khader 2010	0.5	1613		0.05
IMPROVE, Valensi 2009	0.5	52 419		0.008
INITIATE plus, Oyer 2011	0.46	4812	87	127
Levit 2011	2.9	115	0	0
Ligthelm 2009	1.5	149	0	0
Makela 2012	0.5	496		19
Nakashima 2013	0.92	135	3	5
Nobels 2012	0.5	498	6	

Study	Time horizon (years)	Number of participants	Patients with ≥1 SHE	No of events – absolute or mean per patient-year
Pirags 2012	1	1139	28	37 events; 0.04 events/patient-year
PRESENT, Gao 2009	0.23	3697; 4754; 2392; 817		0.04; 0.13; 0.3; NA
PRESENT, Khutsoane 2008	0.50	21 977		0.1
Temizel 2010	1	71		0.06 per patient- month
The 1-2-3 study, Garber 2006	0.31	100 and 68 and 25	3 and 3 and 1	
**Pre-mix human insulin**				
Achieve, El-Naggar 2012	0.08	6323	224	335 events; 0.69 events/patient-year
Gu 2012	0.31 and 0.31	409 and 235		2 and 0
IMPROVE, Shah 2009 a	0.25	3856		0.355
Nobels 2012	0.08	592	4	
PRESENT, Shestakova 2007	0.23	3241	162	0.7
Progens-first-step, Strojek 2008	0.25 and 0.25	482 and 483	1 and 2 patients during first 13-week observation and during second 13 weeks, respectively	2 and 2 episodes, respectively
Temizel 2010	1	69		0.04 per patient-month

	Study	Time horizon (years)	Number of participants	Patients with ≥1 SHE	No of events – absolute or mean per patient-year
	Tentolouris 2013	0.75	159		0.017 episodes/month
**SU**					
	Andayani 2010	0.5	49	1	1
	Aung 2012	1	1043	24	
	Exhype, Pettersson 2011	0.5	430	5 (1.2%)	
	Guo 2013	0.31	390	3	3
	Iványi 2012	2.54	86	2	2
	Klen 2014	0.25	156	0	0
	Obstacle Hypoglycae-mia Study, Kalra 2013	0.23	93; 138; 806; 26	2; 0; 14; 0	
	Panelo 2013	0.5	2370	3	
	UK Hypoglycaemia Study Group	0.73	103		0.1
	Vexiau 2008	0.5	400	16	
